# The 10 m collimated Rowland spectrometer at the MAX IV Veritas beamline

**DOI:** 10.1107/S1600577525005478

**Published:** 2025-07-23

**Authors:** Marcus Agåker, Carl-Johan Englund, Victor Ekholm, Ludvig Kjellsson, Peter Sjöblom, Louisa Pickworth, Johan Söderström, Niklas Johansson, Anirudha Ghosh, Takashi Tokushima, Marco Caputo, Johan Forsberg, Gábor Felcsuti, Pierre Fredriksson, Suleyman Malki, Nial Wassdahl, Conny Såthe, Jan-Erik Rubensson

**Affiliations:** a Department of Physics and Astronomy Uppsala University PO Box 516 SE-75120Uppsala Sweden; b MAX IV Laboratory Lund University PO Box 118 SE-22100Lund Sweden; c Englund Engineering AB, Kättinge 25, 755 92Uppsala, Sweden; Advanced Photon Source, USA

**Keywords:** spectrometer, synchrotron, RIXS, soft X-rays, instrument

## Abstract

A grazing-incidence Rowland spectrometer with a collimating element for resonant inelastic soft X-ray scattering at the Veritas beamline at MAX IV, Lund, Sweden, is presented. The instrument is designed to operate in the 250–950 eV range, providing >30000 resolving power in first order. The inherent temporal resolution of the delay-line detector enables time-resolved experiments on the 270 ps time-scale and also allows for so-called time-gating which significantly improves the signal-to-noise ratio. The instrument is able to rotate around the sample position in the horizontal plane, which enables measurements of the momentum transfer in the X-ray scattering event. The instrument is prepared for installation of a polarimeter to provide full characterization of energy, momentum and polarization of the scattered photons. The design is evaluated using spectral measurements, ray-tracing, vibration and motion measurements.

## Abstract 2

The design and performance of a soft X-ray RIXS instrument at the Veritas beamline at the MAX IV laboratory in Lund, Sweden, are presented.

## 1. Introduction

After the pioneering study (Sparks, 1974 ▸) and early synchrotron-radiation-based experiments (Eisenberger *et al.*, 1976 ▸; Lindle *et al.*, 1989 ▸; Nordgren *et al.*, 1989 ▸; Dallera *et al.*, 1996 ▸; Ghiringhelli *et al.*, 1998 ▸), resonant inelastic X-ray scattering (RIXS) has emerged as a major tool for the investigation of electronic structure and dynamics (Ament *et al.*, 2011 ▸; Schmitt *et al.*, 2014 ▸; Gel’mukhanov *et al.*, 2021 ▸) with a wide range of applications. High-brilliance diffraction-limited storage rings now give the opportunity to refine the technique so that important interactions can be addressed. Notably, high-quality RIXS spectra give access to the coupling between charge, lattice, orbital and spin degrees of freedom, which determines materials’ properties, especially in systems with large electron correlation. Being atom-selective and entirely photon-based, the technique probes bulk properties and can easily be applied to buried structures, liquids, gases and samples at ambient conditions.

The energy resolution is one figure of merit, as the intrinsic widths of the spectral features are limited only by the width of the final states of the scattering process. Therefore, features which are much narrower than the intermediate core-hole states are resolved and it is possible to investigate low energy excitations like magnons and phonons in quantum materials, and soft-mode vibrations in molecular systems. Improvements in resolving power widen the scope of the technique, and in the soft X-ray range this has inspired the construction of large grating-based instruments (Ghiringhelli *et al.*, 2006 ▸; Harada *et al.*, 2012 ▸; Chiuzbăian *et al.*, 2014 ▸), with lengths up to 15 m (Brookes *et al.*, 2018 ▸; Diamond, 2020 ▸; Jarrige *et al.*, 2018 ▸; Singh *et al.*, 2021 ▸; Dvorak *et al.*, 2016 ▸; Miyawaki *et al.*, 2022 ▸), but also more compact systems (Miyawaki *et al.*, 2024 ▸).

Early on, it was recognized that the momentum transfer in the RIXS process can be exploited (Gel’mukhanov *et al.*, 1976 ▸), especially for mapping the dispersion of collective excitations (Ament *et al.*, 2011 ▸). To monitor the RIXS process as a function of momentum transfer, measurements are required at various scattering angles, which also gives the opportunity to investigate the RIXS process beyond the dipole approximation (Söderström *et al.*, 2024 ▸). To accomplish this, it must be possible to rotate the spectrometer around the sample in the scattering plane.

In addition, the transfer of angular momentum in the RIXS process, manifested both in linear and circular dichroism, is taken advantage of in investigations of orbital orientation and magnetic ordering. This becomes increasingly important, especially in combination with high-energy resolution, allowing investigation of new phenomena, as was recently demonstrated in the observation of chiral phonons (Ueda *et al.*, 2023 ▸). Polarization control of incident radiation is achieved using a helical insertion device, and the polarization of the scattered radiation can be analyzed using multilayer reflection near the Brewster angle (Braicovich *et al.*, 2014 ▸).

For the investigation of UHV-incompatible systems, sample-delivery techniques based on liquid beams and cells with ultrathin membranes are typically used. This implies the obvious risk of contamination for the optics, both in the beamline and in the spectrometer, thus protection is required, *e.g.* by differential pumping stages, pin-holes and further membranes to maintain UHV conditions in the optical chambers. Such arrangements must be introduced in a limited space, as the distance between sample and beamline optics must be short to ensure tight refocusing, and the distance between sample and the spectrometer optics must also be short to ensure that a large solid angle is covered.

A versatile RIXS spectrometer must thus have high resolving power, the ability to measure as a function of scattering angle, polarization selectivity and the ability to deal with UVH-compatible as well as non-UHV-compatible samples. In addition, it needs to have high throughput, stability and ease of operation.

**Figure 1:**
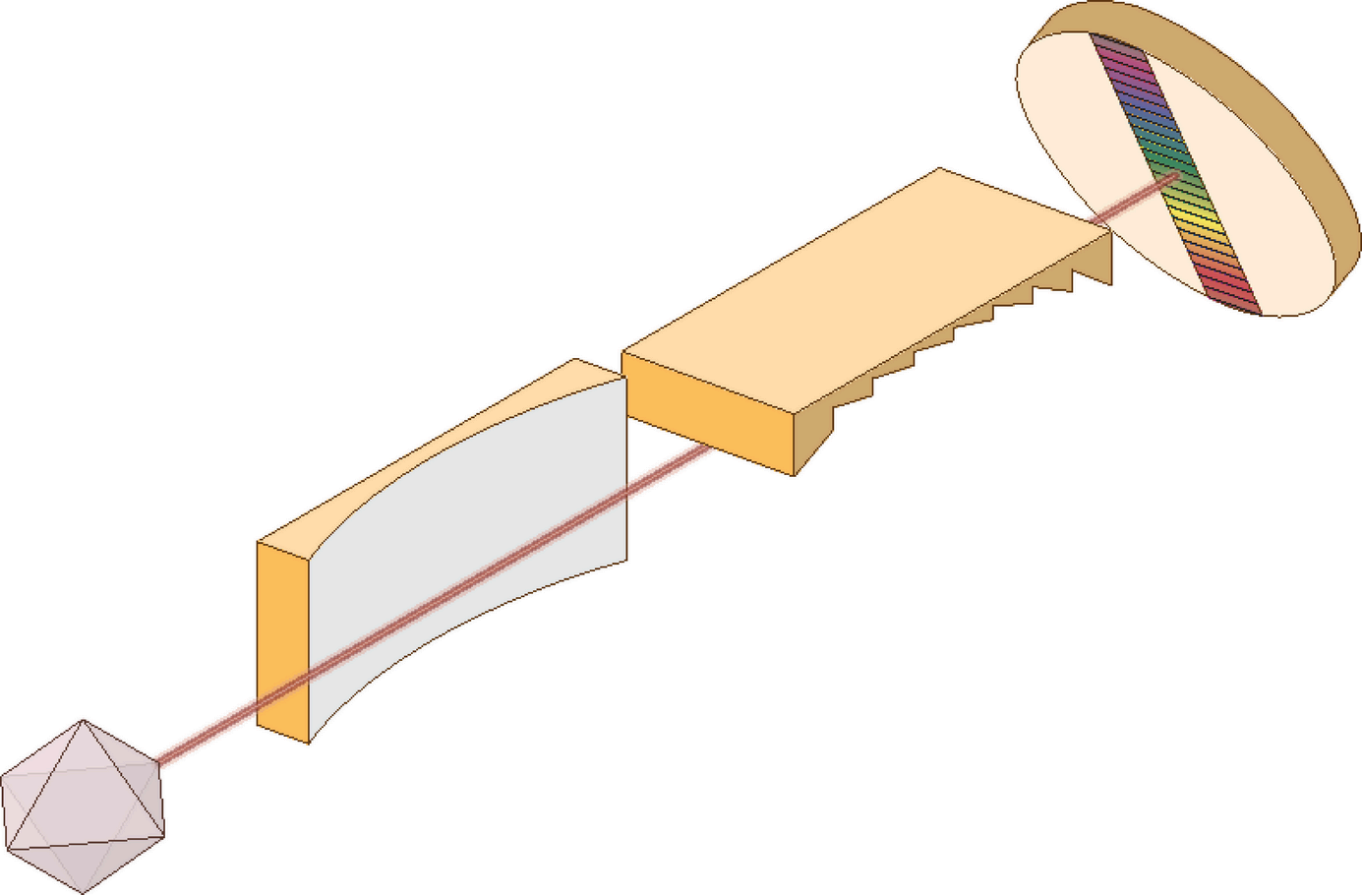
The spectrometer consists of a parabolic collimating mirror in the horizontal plane followed by a cylindrical grating that disperses the incoming light in the vertical plane as a function of wavelength. The dispersion fan is picked up by a detector placed at the relevant position to cover the energy region of interest.

RIXS instruments for the soft X-ray region that aim to fulfill these demands are mainly following the concept shown in Fig. 1 ▸. Incoming light is scattered from the sample in a specific direction, that is collected by an instrument using grazing incidence optics. Optical elements collimate the light, usually in the horizontal plane, while another element disperses the light in the perpendicular plane. The dispersed light is picked up by a spatially resolving detector, recording the intensity as a function of wavelength. The specifics of the optical components and detector type vary between spectrometer designs. In the following, we will describe how the challenges are met in the spectrometer at the Veritas beamline at the MAX IV laboratory (Roberts *et al.*, 2023 ▸).

## 2. Optical design

Before settling on an optical design for the Veritas instrument, a ray-trace survey (Forsberg & Strocov, 2011 ▸) of different optical designs was conducted, primarily focusing on resolving power and throughput. The study also looked at various operation modes needed to cover the desired energy range and achieve sufficient energy windows while maintaining the required resolution.

### 2.1. Varied or constant line spacing

In the study (Forsberg & Strocov, 2011 ▸), varied line spacing (VLS) and classical Rowland (Rowland, 1883 ▸) designs with constant line spacing (CLS) gratings were compared. From a resolution point of view, both designs were found to be similar. Operation of a VLS type instrument over a wide range of energies requires translation and rotation of the gratings in addition to translation and rotation of the detector. Such a complex operation was considered to be a complicating factor, both from a design perspective and from an operator perspective. Also, the more upright focal plane of the VLS designs puts higher demands on the detector resolution than a more grazing-incidence Rowland geometry. This can be mitigated to some extent by using a higher ruling density of the grating, but it is detrimental to transmission. However, in a VLS design, the entrance arm to the grating can be shorter than in a Rowland design, compensating for this to some extent with a larger collection angle for a given optical aperture.

A collimating mirror can also be used to increase the collection angle perpendicular to the dispersion plane. This mirror does require some space in the entrance arm of the grating, and if it cannot be placed inside the experimental chamber it will require space between the chamber and the grating, limiting how close to the experimental chamber the grating can be situated. With this in mind, it was deemed that the difference in the entrance arm length and diffraction efficiency does not give the VLS design much of an improvement in transmission for the cost of mechanical complexity.

In a Rowland type design, the detector needs to accept the incident photons under the same angle as the diffraction angle which, in this grazing incidence design, is of the order of 5–10°. This limits the choice to detectors based on microchannel plates (MCPs). Using VLS type designs makes it possible to angle the focal plane such that it has a 20–30° incidence to the detector surface, enabling the use of X-ray CCD and CMOS detectors. These detectors generally have a higher quantum efficiency than MCPs but they often have a substantial readout time, which somewhat limits the effective exposure time. To improve the signal-to-noise ratio through time gating, time resolution in the ps regime is needed. This also allows for some time-resolved measurements on the same time scales.

Today, there are fast pixel detectors where each pixel is read individually, such as timepix/medipix chips (Ballabriga *et al.*, 2011 ▸). However, these were not accessible at the time of the design of the Veritas instrument.

Considering all these factors, it was concluded that a Rowland type design would not to a large degree compromise the optimal performance of the instrument while offering a simpler operation than a comparable VLS solution.

### 2.2. Collimated Rowland spectrometer

**Figure 2:**
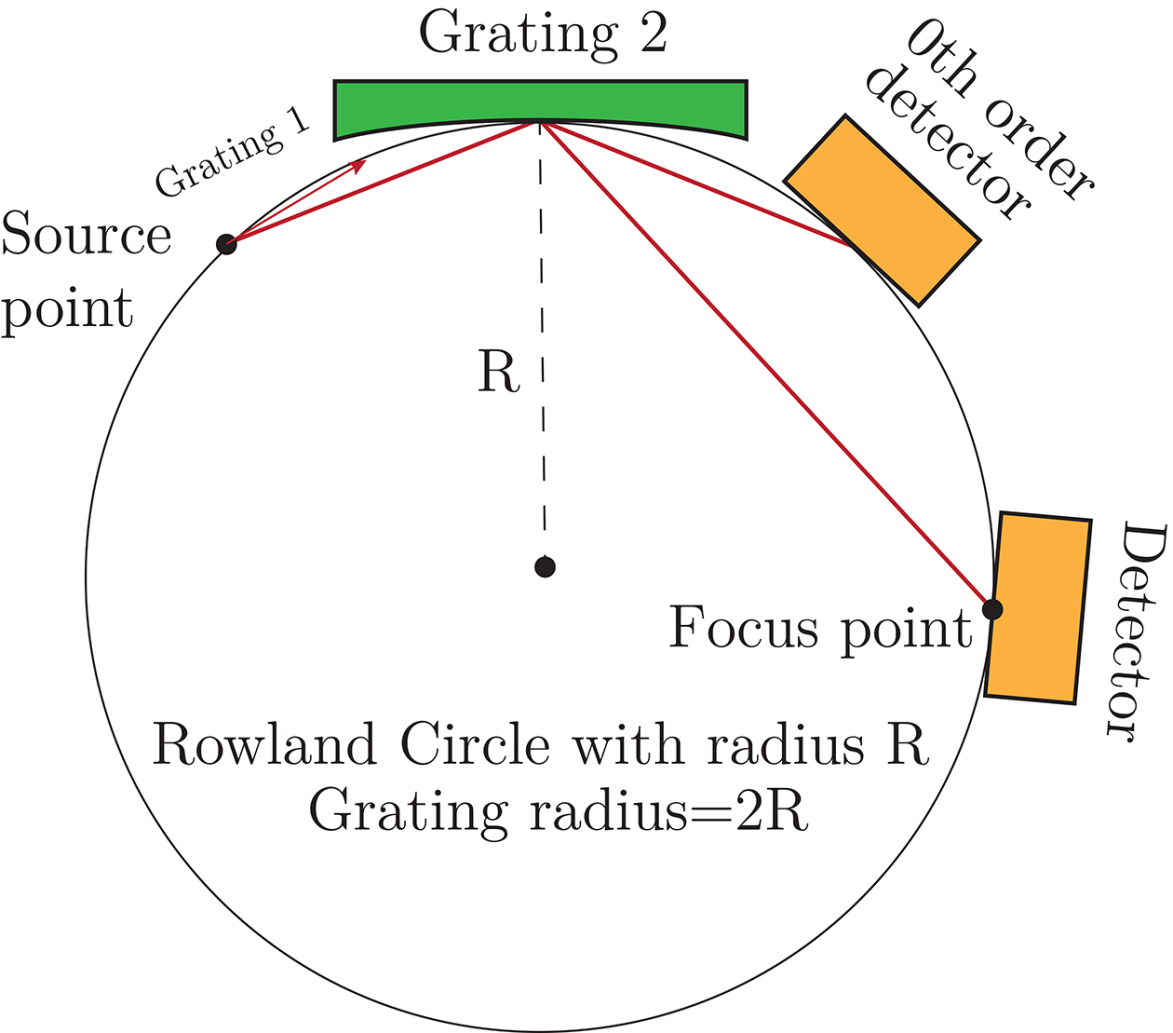
The optical components are placed on a circle with half the radius of the concave grating, with grating and detectors oriented tangentially to the circle. In positive order of diffraction, the exit angle of the beam from the grating is larger than the entrance angle. In the present application, the circle radius is 67 m and the grating and detector are both situated in the 10 m top section of the circle.

In a Rowland type spectrometer (Nordgren & Nyholm, 1986 ▸; Nordgren *et al.*, 1989 ▸) a cylindrical diffraction optics is used to disperse the incoming radiation according to wavelength and at the same time image the source onto the focal plane. In this geometry, the source and detector are placed on opposite sides of the grating on a circle, tangential to the grating, with half the radius of the grating, see Fig. 2 ▸. With the improvements in beamline refocusing the footprint of the X-ray focus on sample can be directly used as a source for the spectrometer optics, eliminating the need for a source-defining entrance slit. This improves the transmission of the instrument, but makes it more sensitive to the beam stability and the beamline refocusing.

The optical design presented for a collimated Rowland spectrometer by Forsberg & Strocov (2011 ▸) was optimized considering a number of parameters set by external requirements at the Veritas beamline. The building limits the total length of the spectrometer to 10 m. In order to improve stability, in accordance with the MAX IV stability requirements, the diffracted light is directed downward to avoid elevated structures at the end of the instrument, which are inherently less stable compared with low structures. The beam height at Veritas is nominally 1.3 m above the floor, which limits the vertical deflection to 1.2 m, leaving a 10 cm gap between the detector surface and the floor at its lowest (maximum deflection) point. The design of the experimental chamber (Englund *et al.*, 2015 ▸) and the requirement to be able to handle solids, liquids and gases prevent any optics from residing inside the experimental chamber, limiting the closest distance to the sample to 500 mm for any optics in the present design.

### 2.3. Collimator

As the spectrometer at Veritas has a length of 10 m, while the detector has a limited useful width of 40 mm set by the vacuum system, a collimator is needed to improve collection efficiency. Due to the large minimum distance to the sample for the optics, the collimator must be about 1 m in length to match the width of the detector (∼30–40 mm) with an incidence angle of 2° at the center of the collimator.

### 2.4. Grating

The need for a 1 m-long collimator plus the minimum distance of the optics from the source puts the closest distance for the grating at 1.5 m from the source. To obtain a reasonable vertical collection angle, this means that the grating also needs to be long. Around 2010, the practical limit was about 160 mm for a ruled blazed grating due to limitations in ruling machinery. With holographic laminar gratings, slightly larger gratings can be used, but these have lower efficiency than ruled gratings. At the time of the Veritas design, a new ruling machine was in development at HZB Berlin (Siewert *et al.*, 2018 ▸; HZB, 2025 ▸) that promised to be able to rule longer gratings. Considering this, the length of the gratings was set to 280 mm. This is larger than the limit where spherical aberrations significantly affect the resolving power in this geometry, but, since the illumination of the grating can easily be controlled, this gives the option to over-illuminate the gratings, losing resolution but gaining transmission.

To cover the full energy range of the instrument, 250 eV to 1000 eV, at least two gratings are needed to reach the desired (>30000) resolving power over the full range in the first order. In addition, the Rowland design makes it easy to operate the instrument in higher diffraction orders, which increases both the energy range and the resolving power of the instrument. The grating ruling densities are optimized to cover the *K* edges of C, N and O and the *L* edges of the early 3*d* transition metals, 250–570 eV, in the first order on the first grating and the *L* edges of the later transition metals, 365–950 eV, in the first order on the second grating. The two gratings can also be used in second order to cover parts of the energy range with higher resolution but with less transmission. To match the length of the instrument and the maximum deflection the two gratings have a radius of 67 m and ruling densities of 1350 lines mm^−1^ and 1750 lines mm^−1^, respectively. The energy range of each grating is limited by the mechanical stroke (2.5 m) of the detector system, *i.e.* the part of the Rowland circle that is accessible to the detector.

To make the mechanical design easier and to simplify operation, the two gratings are placed in a serial mount, where each grating can be left stationary after initial alignment and optimization. In order to avoid shadowing between the two grating positions, one grating is placed slightly above (+0.15°) the horizontal plane, while the other is slightly below (−0.15°). Both gratings have the same radius, so that their positions can be interchanged if needed. Switching between gratings is done by shifting the illumination with a set of baffles, while keeping the grating positions fixed.

The diffracted light from the gratings is spread out along the respective Rowland circle. Due to the large radii of the gratings, it is possible to also access the zero-order reflection focus with a separate detector. This is used to directly monitor the source in parallel to the main-detector measurements, and it can be used as a fluorescence yield detector.

### 2.5. Installed optics

The collimator, produced by JTEC Corp, is a 1 m-long plane parabola with a 1.1 m entrance arm and a 2° incidence angle at the pole. This produces a 34 mm-wide collimated beam that is projected onto the gratings.

The dispersive elements used in the spectrometer are two reflection gratings, ruled on cylindrical mirror blanks from Pilz Optics. These have an optical aperture of 160 mm × 40 mm. There are also longer blanks from JTEC Corp designed for an optical aperture of 280 mm × 40 mm. Ruling of the shorter gratings was done at Helmholtz Zentrum Berlin (HZB), Germany. The shorter gratings have a more relaxed surface quality, as they were acquired as a temporary solution while awaiting the ruling of gratings on the longer, high surface quality, JTEC blanks.

Both the collimator and the gratings are made of single crystal Si(100) substrates and are coated with 30 nm Au, with a better surface roughness than 0.2 nm RMS for the collimator and 0.6 nm RMS for the grating.

The mirror blanks for the polarimeter are produced by Pilz Optics. They are flat Si mirrors with a radius > 10 km and an optical aperture of 30 mm × 30 mm. The multilayer composition and thickness are to be decided and have not been produced at the time of writing. The polarimeter will be discussed in a forthcoming publication.

The optical specifications, and measured values where applicable, are summarized in Table 1 ▸.

### 2.6. Simulated performance of the spectrometer

The expected performance of the RIXS spectrometer can be modeled by ray-tracing. Ray-tracing of the instrument was done in stages during the design process to meet the performance goals for the instrument given the spatial constraints and to set the specifications for the quality and size of the optical components. During the design, *Beam4* (Lampton, 1985 ▸), *RAY-UI* (Baumgärtel *et al.*, 2016 ▸), *XRT* (Klementiev & Chernikov, 2014 ▸), *SPECTRA* (Tanaka & Kitamura, 2001 ▸) and *REFLEC* (Schaefers & Krumrey, 1996 ▸) have been used. Below we present results using *XRT*, *SPECTRA* and *REFLEC*, which include optics in the beamline as well as the spectrometer. This includes the present working conditions of the beamline. This is important since the beamline defines not only the source bandwidth but also the vertical footprint in the experimental focus. These parameters directly affect the performance of the spectrometer.

The ray-tracing simulations are conducted with a total of 24 million rays so that the optical aperture of the collimating parabola is slightly overfilled, which is achieved with a Gaussian source of divergence σ = 40 mrad vertically and horizontally. This divergence overfills the optical surface acceptance of the grating and only 2% reaches the detector surface. This could be improved by underfilling the parabola surface and matching the acceptance of the grating; however, this underestimates the contribution of the parabola surface errors to the final line width at the detector. In reality, the source is emitting into 4π steradians (sr) and the illumination of the grating is fixed by an aperture. To calculate the resolution of the spectrometer, the full width at half-maximum (FWHM) of the line on the detector is used. There is some limitation in the histogram binning widths, given the ray number available, that leads to a numerically driven variation in the results that is not physical; it is expected that the resolution of the instrument varies smoothly over the energy range.

**Figure 3:**
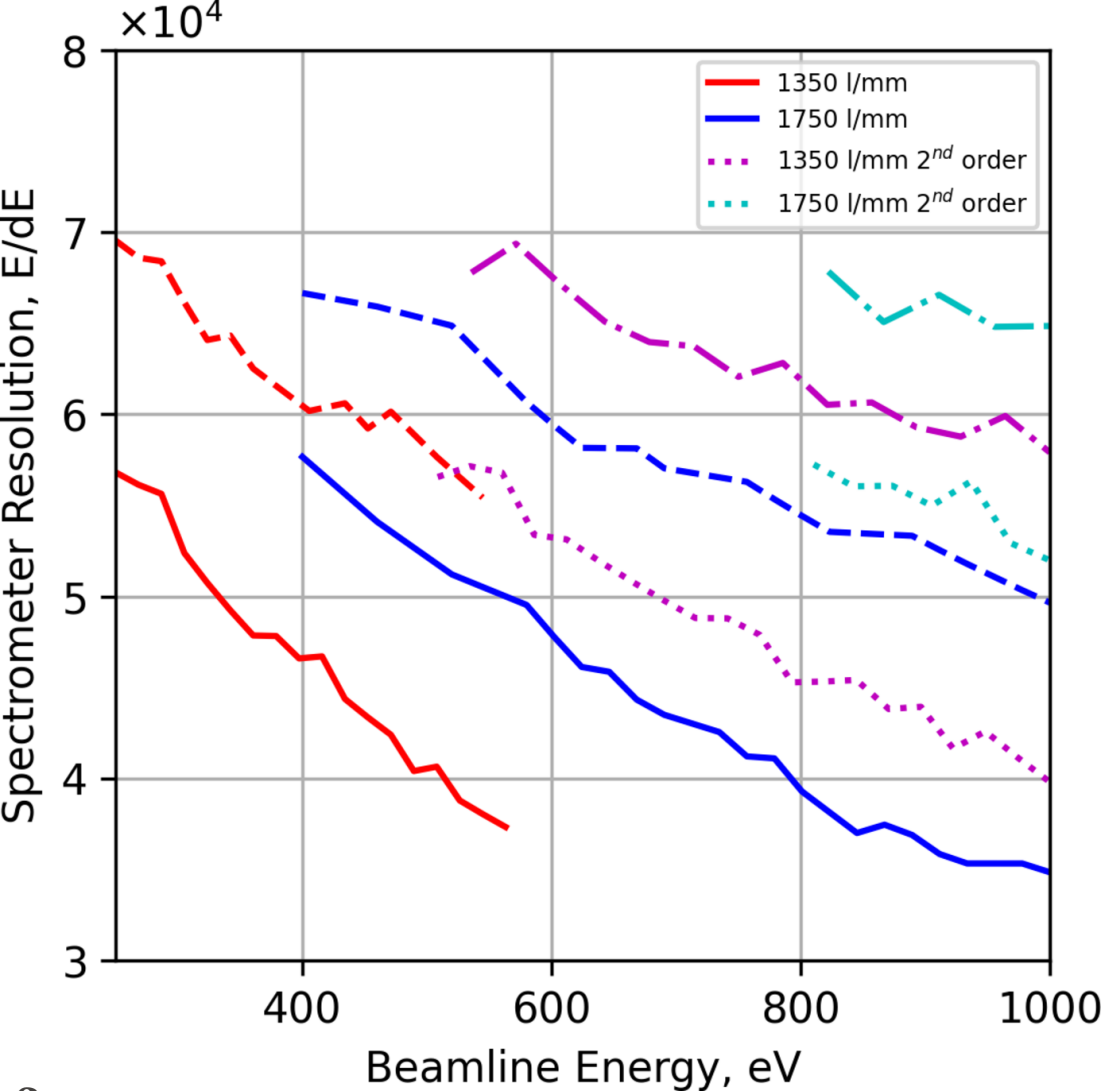
The resolving power of the RIXS instrument for the 1350 lines mm^−1^ (red) and 1750 lines mm^−1^ gratings (blue) over their respective energy ranges, solid line Pilz and dashed line JTEC, with a beamline resolving power of 100000 and source vertical size of 1 µm FWHM.

To determine the contribution to the final resolution of the instrument alone, simulations were first performed with a Gaussian source with a vertical FWHM of 1 µm and a fixed source resolution (*E*/Δ*E*) of 100000, where Δ*E* is the FWHM of a Gaussian energy distribution. Fig. 3 ▸ shows the achieved resolution of the spectrometer for several grating configurations, in first and second order. This case shows more or less the ability of the spectrometer. Solid colored lines are Pilz (shorter and lower surface quality), dashed lines are longer (JTEC) gratings. The second-order resolution is shown in magenta and cyan, the dotted lines are the Pilz gratings, and the dot-dash lines are the JTEC gratings.

As can be seen from Fig. 3 ▸ the potential resolving power of the instrument with the JTEC gratings is of the order of 60–70 K at low energies and drops off to about 50 K at the high energy cut-off. However, the currently installed gratings from Pilz Optics are limited to deliver from 55 K to 35 K due to higher meridional slope errors, see Table 1 ▸.

**Figure 4:**
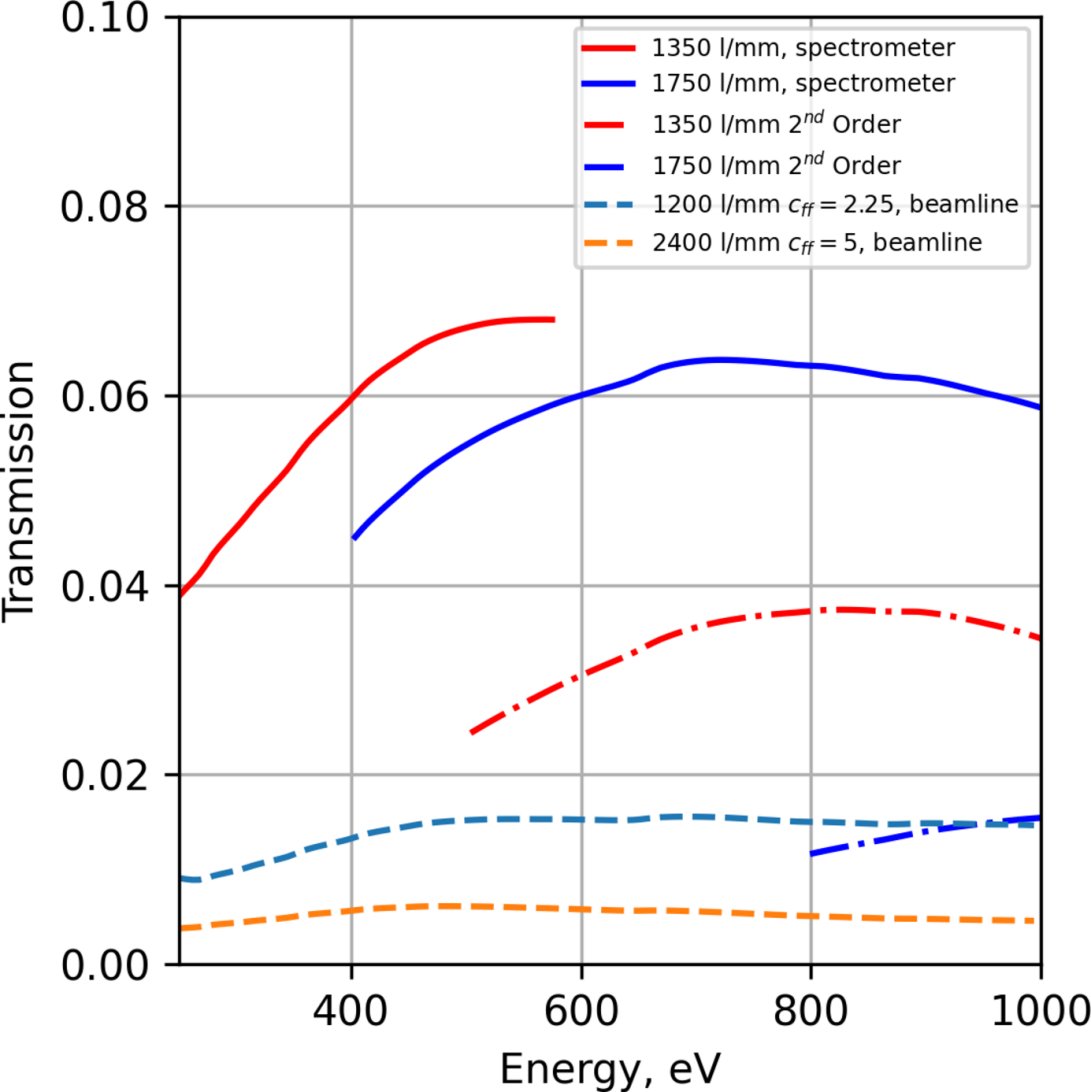
The transmission of rays hitting the first optical element of the RIXS instrument for the 1350 lines mm^−1^ (red) and 1750 lines mm^−1^ (blue) gratings in first (solid) and second (dotted) order of diffraction. The Veritas beamline transmission for the 1200 lines mm^−1^ (light blue dash) and 2400 lines mm^−1^ (yellow dash) gratings.

The transmission efficiency of the spectrometer is shown for the first and second order in Fig. 4 ▸ for the two ruling densities. For comparison, the efficiency of the beamline for two configurations of the monochromator is also shown when the exit slit/secondary source vertical slit size is set to 20 µm. When the collimated monochromator of the beamline is set to 1200 lines mm^−1^ with a fixed focus condition *c*_ff_ = 2.25 (Petersen, 1982 ▸), this represents a high-flux operation, where the energy bandwidth is increased. In contrast a 2400 lines mm^−1^ grating can be used with *c*_ff_ = 5 which gives a high energy resolution configuration. The monochromator of the beamline can further increase the *c*_ff_ value which will increase the beamline resolution under nominal operation conditions (Sjöblom *et al.*, 2020 ▸); however, the transmission of the monochromator decreases with an increase of *c*_ff_.

Fig. 4 ▸ does not account for source emission that does not interact with optical surfaces. The solid-angle acceptance limits the efficiency when the source has a quasi-isotropic angular distribution. In the case of the spectrometer, the horizontal collection is limited by the parabola length, and the vertical collection is limited by the grating length. The installed Pilz gratings accept a solid angle of 7.1 × 10^−5^ sr (the collimator accepts 1.01 × 10^−3^ sr if fully illuminated); the unruled longer gratings will accept a solid angle of 1.33 × 10^−4^ sr assuming that the width of the ruling area remains the same. The primary advantage of longer gratings is improved collection efficiency, but in this case the longer gratings do also have better surface quality, resulting in higher resolving powers.

However, obtaining a beamline resolution of 100000 is not a practical operational setting primarily due to the loss of available flux. A *c*_ff_ = 12 and 2 µm exit slit would obtain this resolution based on the installed optical components of the beamline. Realistically, the beamline is operated with a lower resolving power which influences the performance of the combined beamline–spectrometer in RIXS experiments.

**Figure 5:**
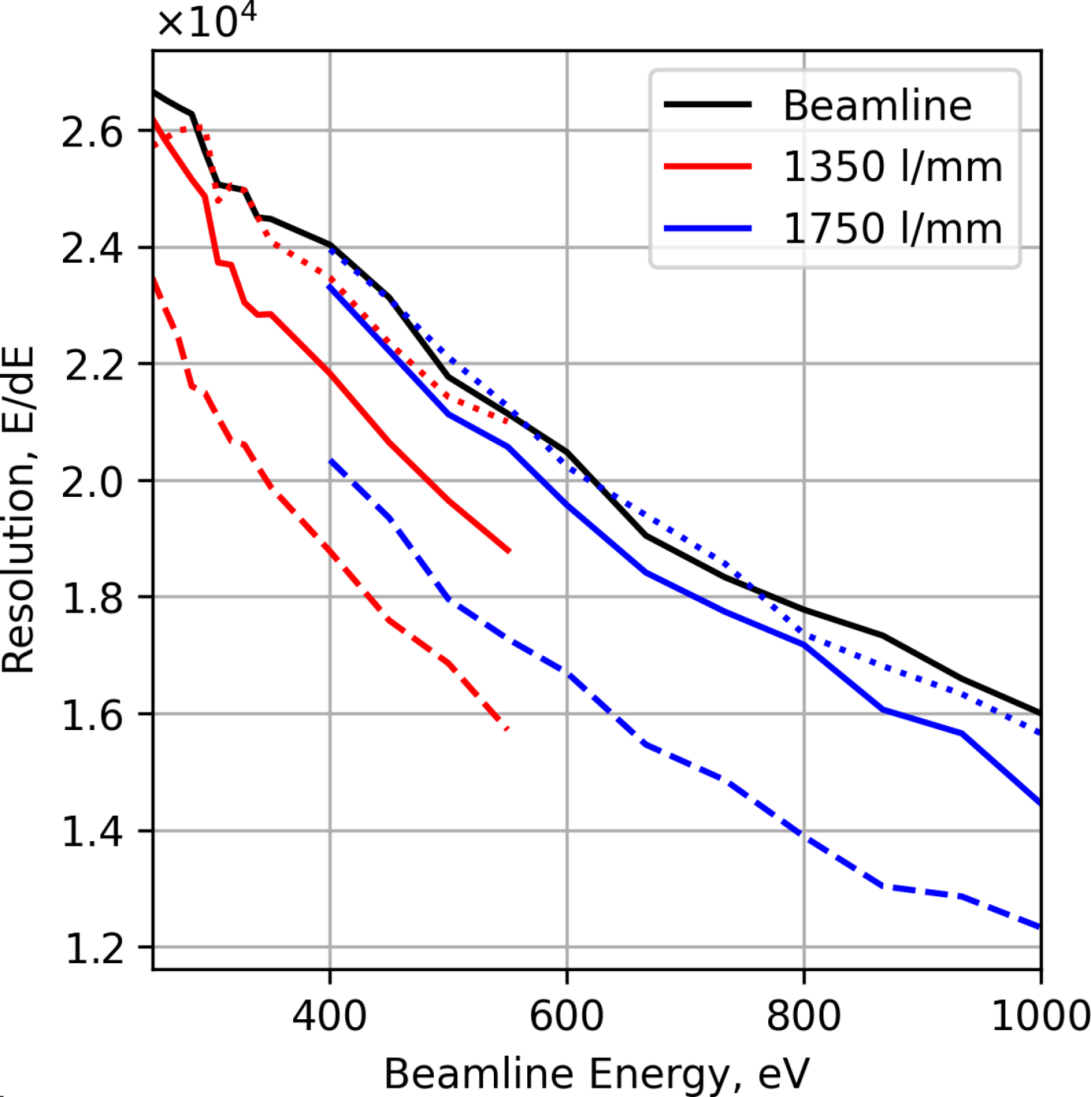
Resolving power of the RIXS instrument in combination with the beamline using the 1200 lines mm^−1^ gratings, 20 m exit slit and *c*_ff_ = 2.25. The 1350 lines mm^−1^ (red) and 1750 lines mm^−1^ (blue) gratings are shown over their respective energy ranges for the Pilz (solid), JTEC (dashed) and apertured JTEC (dotted) gratings. Beamline resolution alone shown in black for reference.

Figs. 5 ▸ and 6 ▸ show the resolution of the spectrometer with the beamline resolution calculated assuming a 20 µm exit slit with the 1200 lines mm^−1^ grating with *c*_ff_ = 2.25 (high flux) and with a 2400 lines mm^−1^ grating with *c*_ff_ = 5 (high resolution). Simulation of the beamline was performed in *XRT* with 4800000 rays and a full ideal linear undulator simulation operating on the harmonic (*i.e.* highest flux on axis). The source of the beamline then has a Gaussian-like spatial and angular ray distribution. The energy bandwidth was chosen to overfill the imaged spectrum on the exit slit, where the vertical opening of the exit slit determines the resolution and source size for the spectrometer. For a fixed exit slit size the resolution of the beamline decreases with energy, while the source size for the spectrometer remains the same over the energy range, ∼1.5 µm FWHM.

The resolving power observed in the spectrometer is to a large extent limited by the beamline. Figure error differences between the spectrometer optics make no difference in this regime; rather, the longer gratings are more susceptible to spherical aberration with this large beamline bandwidth, making them seemingly worse. This can be addressed by limiting the illumination of the longer gratings to below the aberration limit as seen by the dotted lines in Figs. 5 ▸ and 6 ▸ representing the JTEC gratings limited to the same illumination area as the Pilz gratings.

Another important factor for the resolution of the spectrometer is the vertical footprint in the interaction region, as the overall resolving power is a combination of source bandwidth and imaging of the source size. Fig. 7 ▸ shows the resolution in the spectrometer as a function of source size with a fixed beamline resolution of 100000 at 529.4 eV. The source size has a minor impact on the overall resolution as long as the size remains smaller than 5 µm, especially for the currently installed Pilz gratings.

In the case of Veritas, the performance of the setup is thus limited by the beamline performance, regarding what can realistically be achieved in terms of resolution and intensity in RIXS experiments.

### 2.7. Measurements

The beamline has primarily been operated with the 1200 lines mm^−1^ grating in the monochromator, thus limiting the ultimate resolution but providing higher transmission needed for the alignment process. This means that the spectrometer has only been tested towards this beamline performance and, as can be seen from the ray-trace, the overall resolution achieved in the instrument is dominated by the beamline under these circumstances. Figs. 8 ▸(*a*) and 8(*b*) show results of vertical edge scans through the beam at different positions in front of the spectrometer. At best focus the FWHM of the beam is ∼1 µm. Fig. 8 ▸(*c*) shows the elastic peak, measured at 468 eV using the 1200 lines mm^−1^ grating in the monochromator with a 5 µm exit slit and *c*_ff_ = 2.25. The resulting FWHM according to a Voigt fit is 15.8 channels (28 meV) on the spectrometer detector, corresponding to a resolving power of 16700.

The design of the instrument allows for continuous measurements of the signal during rotation around the sample point. Fig. 9 ▸ shows the elastic peak position in the dispersive direction on the detector during a rotation of the spectrometer in the horizontal plane, corresponding to a variation in scattering angle from 42° to 143.2°. There is a slight drift of the peak position within a ∼50 channels band during motion. This can be compared with the peak width of 15.8 channels FWHM as seen in Fig. 8 ▸. This means that for each scattering angle the energy scale needs to be reset. However, the width of the peak is unaffected by the motion and hence the overall resolution is conserved. For an example of measurements carried out with the instrument, utilizing the high resolution and *Q*-measurements, see Söderström *et al.* (2024 ▸).

## 3. Implementation

The different sections of the spectrometer are illustrated in Fig. 10 ▸ both as a simplified CAD-model and the actual installation.

### 3.1. Detectors

The Rowland design requires a detector capable of operating in grazing incidence mode, as described in the optical design section[Sec sec2]. In this instrument, an MCP-based detector with a delay-line anode is used, due to their performance at grazing incidence angles, short readout time and time-resolving capabilities. One diagnostic MCP-detector with a fluorescent screen coupled to an optical CCD camera is used at the zero-order diffraction position of each of the two gratings in the spectrometer. An overview of the detectors is given in Table 2 ▸.

#### 3.1.1. The delay-line detectors

The main detectors (recording spectra) are two DLD8080 delay-line detectors (DLDs) manufactured by Surface Concept GmbH. These are equipped with two chevron mounted 80 mm MCPs with a pore size of 10 µm. With the readout electronics, the detector has a cell size of 10.1 µm and a spatial resolution of 50 µm.

The inherent time electronics also give a time resolution of 27 ps per channel and an overall time resolution of 270 ps. The timing of the delay-line readout is synchronized to the machine clock of the synchrotron, and by utilizing this all counts registered between the light pulses can be discarded. This effectively reduces the number of dark counts by 90%, allowing experiments to be done on weak signals.

Output from the DLD is a stream of *x*,*y* and two time coordinates (local time in relation to the bunch clock and global time, *i.e.* time since the start of the measurement) for each event recorded in the detector. These coordinates are then processed in a custom software described bellow.

#### 3.1.2. Zero-order detectors

The zero-order detectors, used to monitor the source position in relation to the gratings, are from Tectra GmbH. These are 50 mm MCPs with a pore size of 12 µm and a P43 fluorescence screen. The screen is viewed by a Baseler aca2440-20gm combined with a lens imaging about 50 mm of the fluorescent screen. In this configuration, each pixel corresponds to ∼20 µm. The detector image is processed by an event-finding software that converts the image to an (*x*, *y*, *t*) coordinate that can be processed in the same software as the DLD stream.

#### 3.1.3. Software and readout

A custom software was developed for readout and visualization of the spectrometer data (Kjellsson, 2015 ▸). The DLD is read in a streaming mode, with the counted photons continuously integrated both as a 2D detector image and a spectrum displayed to the user, see Fig. 11 ▸(*a*). Data are received as a stream of *x*–*y* coordinates as well as an arrival time both in relation to the bunch structure (picoseconds) as well as in a global frame (unix time stamp in milliseconds). A number of other parameters can also be monitored for each event such as undulator settings, monochromator energy, ring current, sample position, spectrometer scattering angle, spectrometer DLD position, *etc*. This allows for a filtering of the data on these parameters, creating spectra limited to a specific range. One such standard filtering is to use the arrival time (in relation to the bunch structure) for time gating to only use events arriving together with the bunch and discarding events outside this timing window, see Fig. 11 ▸(*b*). Once filtered in time with respect to the bunch arrival time, the data can be further treated by removing hotspots and correcting for optical artifacts such as bending of lines before summing to a spectrum. Tools are available to display the incoming data as a function of time in waterfall plots or as maps based on a specific parameter such as incoming energy or scattering angle. To assess data quality, the user can perform peak-fitting and follow spectral changes such as drifts or sample damage by comparing overlays of spectra integrated from different time regions in the measurement.

### 3.2. Mechanical design

MAX IV identified early on the importance of a stability policy to ensure that the eigenfrequencies of components influencing resolution, spot size and beam position should be above 55 Hz, as far as practically possible. The purpose is to avoid a situation where background vibrations, which are always present, excite eigenmodes of the equipment, which in turn could influence the characteristics in a negative way.

To quantify background vibrations early on, a green field survey was carried out during the MAX IV construction phase of the Brunnshög site in North-East Lund by the Norwegian Geological Institute (Rothschild, 2009 ▸; Agåker *et al.*, 2020 ▸). The report shows that most ground vibration amplitudes are in the 40–60 nm range and occur between 5 and 20 Hz. With this information, a 55 Hz limit was imposed, which gives a safety margin both for the occurrence of natural vibrations and for the design of mechanical systems to avoid too low eigenmode frequencies.

In this section, the mechanical design and the philosophy behind the design choices are discussed, with focus on stability and functionality.

#### 3.2.1. Granite foundation

As a mechanical foundation of the spectrometer, a granite block (1550 mm × 980 mm × 830 mm, L × W × H) of 3 tons, under-grouted to the floor, is used. To simplify access to the analysis chamber (Q-chamber) (Englund *et al.*, 2015 ▸), a cutout is located just under its center position. The foundation is also made to double as a base for the M4 beamline refocusing mirror (Agåker *et al.*, 2020 ▸). By using the same base support for the M4, sample manipulator (Agåker *et al.*, 2021 ▸) and spectrometer arm, stability is aided, as seen in Fig. 12 ▸.

#### 3.2.2. Focal point adjustment platform

On top of the granite block is a cast stainless steel plate assembly. This assembly consists of two fixed base plates on each side of the cutout in the granite block and a six-axis adjustable top plate that houses the lower part of the spherical axial roller bearing, holding the spectrometer arm and defining the rotation point of the instrument, see Fig. 12 ▸.

The top plate rests on a three-pillar support with both lateral and horizontal adjustment capabilities. This gives the possibility of aligning the spectrometer focal point to the beamline focal point defined by the M4 refocusing mirror. Once aligned, it can be locked and secured in place laterally by three brackets.

The top plate is also made to house the stand for the experimental chamber and the stand for the sample manipulator. By sharing that mechanical reference, the experimental chamber and sample manipulator automatically follow the spectrometer focal point position when this is adjusted.

These three items—spectrometer, experimental chamber and manipulator—are partially decoupled from each other and only use the top surface of the platform as a common reference. This decoupling minimizes stability interference between the different components. It also makes the system modular and facilitates future upgrades or modifications.

#### 3.2.3. Axial spherical roller bearing

One of the spectrometer’s basic requirements is that it should be capable of continuous scattering-angle adjustment (rotation around its focal point) in the horizontal plane.

The focal position of the instrument is defined by an axial spherical roller bearing (bottom part mounted in the adjustment plate and top mounted in the spectrometer arm), see Fig. 12 ▸. The advantage of using this type of bearing is that it can be used both as focal and mechanical reference point of the instrument, provided that the roll point of the bearing is set up to coincide with the focal point of the beamline and the instrument optics. This enables all of the instruments’ optics and detectors to aim at the same point independent of both scattering angle and errors in the flooring of the facility. This can be seen in Fig. 9 ▸, where the motion of the elastic peak over the rotation represents a difference in optical path length of approximately 0.5 mm.

#### 3.2.4. Experimental chamber

In order to maintain a vacuum around the sample during spectrometer rotation, a low-profile, single-bearing rotation chamber with a 120° motion angle was developed (Englund *et al.*, 2015 ▸). Spring-loaded seals, differentially pumped in two stages, are used to achieve UHV performance during rotation.

#### 3.2.5. Spectrometer arm

In order to fulfill the MAX IV stability policy, performance specifications and budget limitations, the spectrometer main frame is designed to be as stiff, light and simple as possible without compromising critical component serviceability and access.

Since the concept of using a spherical bearing for rotation, with the optical source at the bearing roll point, is a central part of mechanical design, most other mechanical design considerations were taken with this in mind.

The downward slope of the beam path in the optical design also set constraints on the design of the spectrometer arm. After considering different options, a truss-based design was chosen, as seen in Fig. 13 ▸. The base truss sections are made of cross-braced 100 × 50 × 3 VKR steel piping. The truss structure itself handles the actual weight and span of the instrument and internally gives room for the spectrometer beam path, vacuum chambers and other needed components. To handle possible torque loads that could be introduced, by, for example, breakaway torque in the bearing, a full-length, part triangularly boxed, closed section was integrated in the lower part of all of the framework sections.

To aid in manufacturing, handling, and to make it possible to perform future upgrades, the 10 m frame is divided into three major pieces that are firmly bolted together, see Fig. 13 ▸(*a*).

The first part is a two-piece stainless steel cast nose, the front end with its tip housing the upper part of the spherical bearing. The nose acts as a base for both the collimator and the gratings, giving them the same mechanical reference. Using casting technology in this section aided in simplifying a design that by necessity was more complicated than the rest of the framework due to space constraints around the bearing and experimental chamber. This meant that an advanced shape could still be obtained at a reasonable cost, Figs. 12 ▸ and Fig. 13 ▸(*a*), part 1.

The second, or intermediate, part is a welded truss structure with a square cross section. This part houses the zero-order detectors and a mechanism for changing the spectrometer main beamline path.

The third section is also a welded truss structure similar in conceptual design to the second one. On top of this truss sit the linear guides for the detector cradle.

All together, with the downward beam slope that was chosen, the height, weight and volumetric spread came down to a minimum. Thereby a very low center of gravity was achieved, see Fig. 13 ▸.

In addition to the spherical bearing, the arm is also supported by two peripheral support points located on an A-arm truss positioned vertically and normal to the main frame, in the middle of the detector travel path, as seen in Fig. 13 ▸(*b*). The A-arm is further supported by two triangle arms, connecting the midsection of the frame to the support feet as seen in Fig. 13 ▸(*a*), to prevent vibration modes in the frame.

#### 3.2.6. Air feet

In parallel with the bearing solution, an almost frictionless air cushion system was implemented. This makes it easy to move the instrument, which weighs close to 3 tons. What has previously normally been used for this type of spectrometer are radial rail guides with extreme requirements for absolute flatness of the floor and other plane references. Such solutions tend to be expensive and bulky. To give the spectrometer a stable and well defined three-point position, the back end of the frame needs two more support points. They consist of lift modules, the red device in Fig. 13 ▸, actuated by compressed air that doubles as both support and lifting devices. Once actuated, the spectrometer can rotate around the main spherical bearing to the desired position, after which they are retracted. Two extra landing feet handle the actual operational position on the floor, visible next to the red cushions in Fig. 13 ▸(*a*). The floor surface requirements needed for operating these devices does not require any special polishing, which eliminates the need for any special floor preparations.

#### 3.2.7. Grating selection aperture

The two gratings are placed in series and at fixed positions to simplify the design and manufacturing. To restrict both vertical and horizontal illumination, an in-line baffle setup is used. Due to space constraints, a 24 mm thin design with two vertical and two horizontal baffle blades facing each other was developed. The actuators can move independently of each other, covering the full width and height of the gratings. The actuators are all facing upward because of space limitations and are all equipped with absolute encoders. The aperture has a twofold function. It defines the beam cross section, effectively choosing which parts of the collimator and grating optical surfaces are illuminated. By moving the aperture, either of the two gratings can be selected.

#### 3.2.8. Optics

Another challenge is to position and fixate the up-to-1 m-long optical elements, see Fig. 14 ▸, without risk of collision and any resulting surface deformation. Common to all optical elements is a design developed to fix the optics in a position as stress-free as possible. As a result, all optical elements are equipped with through holes, close to the back side. In these holes, rods with spherical contact surfaces are inserted. These support rods are then seated in their respective cassette or cradle. This allows the suspension forces to be optimized for minimal deformation. FEM (finite element method) analysis was used to find the final positions of the contact surfaces with respect to a minimal deformation. For the large collimator element, placement in *Z* (along the beam path) and *X* (horizontal) direction is controlled by two of these rods. For the *Y* (vertical) direction, a spring-loaded mattress-like solution was designed. The collimator is placed on its side, defined and horizontally supported by three fixed steel balls. To counteract the gravity force, 23 spring-loaded steel balls are evenly spread in a pattern adjacent to the three fixed supports. Support of the weight is thereby also evenly distributed over a large area, avoiding point-induced deformations of the element.

For the grating optics, a stepper motor-driven cassette solution with high-precision screws was developed, which enables independent adjustment of roll and pitch *in situ* of the gratings. Gratings are hung in a cradle, see Fig. 15 ▸, by the special support rods. Together with the rest of the necessary adjustment and position monitoring hardware, these form the base cassette module. The cassettes in turn mount in a receiver support that has two sets of V-block guides mounted on a plane corresponding to the chosen grating angles. These guides act as *Z* and *X* base reference points. To enable the mandatory fine-tuning of the grating roll and pitch, three piezoelectric-driven micrometre screws were mounted in a triangular pattern seated in the V-blocks. These form a three-legged pillar stand and each screw can be adjusted ±3 mm to optimize the position of the gratings.

To minimize the tip friction and load on the piezo screws, spring-loaded support pins are used to counteract the grating weight. The modular cassettes make it possible to relatively easily change to gratings with both other sizes and performance, without having to redesign the adjustment mechanism.

The temperature-dependent change in length of the collimator itself and the grating receiver is handled by a combination of rotational and linear flexure solutions, designed to minimize surface deformations. This also eliminates some of the mechanical stress that could be induced by possible errors in machining, dimensioning or mounting.

#### 3.2.9. Energy selection cone

The cross section of the beam from the gratings increases with distance due to the energy dispersion. To keep the spectrometer compact, an adjustable cone is mounted to select the energy range of interest transmitted towards the detector. The cone attaches to a hinge, and the outlet port position is coordinated to the selected energy range. In this way the vacuum tube and bellow diameter can be kept small while still allowing the full spectrum, corresponding to the detector surface, through.

#### 3.2.10. Zero-order detectors

The two zero-order detectors are housed in two separately positioned chambers above the main beam path. Each detector sits on a linear table that allows height adjustments of the detector (±6 mm). The linear table is mounted on a cylindrical surface, the axis of which coincides with the detector surface. This allows an adjustment of the initial incidence angle in the range 2–8°. All the adjustments of the zero-order detectors are manual which means that they cannot easily follow the fine adjustments of the gratings, and must be manually adjusted once the final positions of the gratings are set.

#### 3.2.11. Beam transport bellow

To handle the fairly long travel distance between the two extreme positions of the detector sled, a scissor-supported bellow is used. The scissors’ upper and lower arm lengths sections are slightly different, resulting in a theoretically upward bow at stretched rest. With the inevitable play present in the scissors’ moving joints, it ends up as a straight, linear travel when mounted and subjected to its own weight and carrying loads.

#### 3.2.12. Detector sled

The detector system is positioned on a sled-like carrier riding on SHS-type rails mounted on top of the back end of the frame. This allows for the detector movement in the beam direction (*Z*). To follow the Rowland circle, a vertical (*Y*) axis adjustment screw works in conjunction with the *Z* motion. The detector incidence angle is adjustable between 0 and 10° via rotation of its chamber. All critical components are situated in between the rails for stability reasons. The sled also carries a small ion pump and valve chamber, making it a somewhat independent vacuum system, enabling easier detector servicing and replacement. The detector electronics and computers necessary for its function are mounted on the rearward portion of the sled, see Fig. 16 ▸.

There is also a secondary detector system and a chamber to hold a set of multilayer mirrors for a polarimeter. This polarimeter chamber has the ability to rotate around the center of the main detector housing, forcing its center to have a consistent distance of 400 mm to the main detector position. To synchronize the polarimeter chamber and detector with both the beam path and the main detector position, the polarimeter chamber is also mounted on a vertical adjustment screw. The polarimeter detector is also rotated around the polarimeter chamber, with a fixed distance of 400 mm. It can be positioned between 90° and 45° relative to the beam with an adjustable incidence angle of 0–10°. Inside the polarimeter chamber sits an inline mirror cassette with one clear straight through path and three optional reflecting mirrors. This cassette is mounted on a rail transverse to the beam (*X*) which enables both mirror selection and redirection of between 0 and 100% of the incoming light to the polarimeter detector. To match the mirrors with the chosen polarimeter-detector position, the cassette is made rotatable around its transverse linear movement in the center of the polarimeter chamber.

## 4. Mechanical stability

When designing the spectrometer arm, special care was taken to ensure that no eigenmodes below 55 Hz were introduced, as far as reasonable, in line with the MAX IV policy discussed above.

This section reports on vibration measurements conducted on the spectrometer arm and its components, which were carried out at various times between 2022 and 2024 in order to also capture any long-term variations. The aim of these tests was to characterize the detector arm’s sensitivity to environmental vibrations, understand the underlying mechanism, and assess the impact on experimental quality. Other factors influencing mechanical stability, such as fluctuation in ambient temperature or long-term drifts, have not yet been investigated.

The reported pitch values were approximated from simultaneous measurements with two Wilcoxon 731A seismic accelerometers located on the grating at a known distance (∼50 cm apart). Leaky integration was applied after bandpass filtering 4–100 Hz to the obtained time series, which were further filtered with an exponential time filter SLOW with 1 s time constant according to IEC 6672-1.

### 4.1. Background vibrations

A laser interferometer was used to measure the background vibrations that are present without any induced external sources (as far as possible). Measurements were conducted at several locations on the arm to measure the naturally occurring vibrations in the system as well as the eigenmodes. The setup was based on a Polytech laser vibrometer with the OFR-500 controller.

The laser was placed on three different sections: detector area, mid-section and rotational center measuring in the horizontal plane, see Fig. 13 ▸. For each of those sections, several measurement points were selected to give relative results to each other. In Fig. 17 ▸ the Fourier transform analysis of the vibrations is shown. Three strong resonances are seen at relatively low frequencies, 14 Hz, 18 Hz and 22 Hz, neglecting the smaller 11 Hz sideways bending similar to the 14 Hz motion. There is also a peak at 20 Hz, but this is only seen with the interferometer and not with the vibrometers and is potentially from the laser head support. The 14 Hz mode is the strongest and the following resonances are all much smaller and for higher frequencies they are all fast falling in amplitude.

### 4.2. Experimental modal analysis

To identify the modes seen in the frame, an experimental modal analysis was carried out. The test was carried out according to the roving hammer technique, as described, for example, by Ewins (2009 ▸), with a hammer instrumented with a Dytran 1053V2 force sensor and B&K 4507-B-006 structural accelerometer. The measured transfer functions were analyzed with MATLAB’s modalfit routine.

The identified vibration modes are identical to the lowest vertical bending, the lowest horizontal bending and the lowest twisting eigenmodes of a beam with similar bending stiffness and mass properties as the spectrometer arm’s truss structure. These vibration modes are illustrated in Fig. 18 ▸.

One should note that the measured mode shapes are incomplete in the sense that only the truss structure was included in the measurement while other parts such as the manipulator and the cast iron frame were ignored. This gave rise to additional peaks in the response spectra, which are driven by resonances in other parts of the full structure. This is exemplified by the 11 Hz peak in Fig. 17 ▸, which is identical to the shape of the 14 Hz mode.

Excitation of the 18 Hz vertical bending mode induces pitch rotation and vertical motion of the grating and vertical motion of the detector. This frequency dominates the vibration response under ambient conditions, as shown by the other reported measurements; consequently it has the potential to influence the resolution of the instrument.

### 4.3. Induced vibration test

To see if the noted eigen-modes influence the performance of the instrument, vibrations were induced with an electrodynamic shaker, while the line width, in channels, on the detector was recorded as a function of frequency and amplitude. Detector data were recorded simultaneously with the frequency of the shaker and correlated to vibration amplitudes based on timestamps (the valve was opened before and after the measurement which caused a vibration peak and can be seen in the detector data).

The measurements, see Fig. 19 ▸, were carried out with sinusoidal force excitation at 1, 2 and 3 V amplitude. Sweep excitation was first performed to find a predominantly horizontal and a predominantly vertical vibration mode to use. The measurements were then performed at these two frequencies in order to maximize the vibration energy transferred to the structure. The shaker was oriented in the transversal plane at an angle (elevation angle about 30°) to be able to excite both horizontal and vertical vibrations simultaneously without being remounted.

The test was repeated with extra supports at the middle of the arm to see if it could potentially improve the detector data quality. Without support in the mid-section the motion did not affect the optics and detector located at the two ends of the spectrometer. With a support in the center, the ends of the spectrometer were more susceptible to vibrations and a broadening of the detector line was observed.

As the accelerometers record the amplitude, the difference in amplitude from two time synchronized accelerometers with a known distance between them gives the pitch. On the detector, a less sensitive triaxial accelerometer measured in three perpendicular directions and an additional, more sensitive (albeit redundant), seismic accelerometer measured vertically.

During the test occasion, the effect of excitation of the horizontal mode was found to be negligible, while excitation of the vertical mode caused obvious deterioration of the detector data quality, going from 28 channels FWHM to 100 channels FWHM at full energy transfer. An extrapolation of a fit to the measured points shows a smallest FWHM of 17 channels at 0 induced pitch at the grating. This coincides rather well with the smallest measured FWHM as seen in Fig. 8 ▸. Correlating the measurements with simulations, the line broadening is probably driven more by position of the detector than other factors, like focal shift.

### 4.4. Disturbances due to pallet truck vibrations

The Veritas spectrometer is located in close proximity to the connection between the buildings of the 1.5 GeV and 3 GeV rings of MAX IV, and the fact that this area experiences considerable internal traffic raises concerns about potential vibrational disturbances affecting Veritas, as seen in the measurements with induced vibrations.

To evaluate the worst-case scenario, a 3 ton pallet truck was repeatedly driven over the expansion joint between the two buildings to induce floor vibrations, while the associated response on the spectrometer arm was measured. The results in Fig. 20 ▸ reveal that floor vibrations are amplified in the arm structure (in the absence of vibration sources on the arm), and that these vibration peaks can reach multiples of the undisturbed levels. The typical ambient vibrations measured on the grating is 10 nrad, which is amplified three times to 29 nrad when the truck passes by. Most of the vibration energy is centered in a narrow band around 18 Hz which indicates resonant amplification. The 18 Hz mode is also the one mode observed to significantly influence the resolution of the instrument raising concerns for the overall performance of the instrument.

From Fig. 19 ▸ induced grating pitch vibrations of 500 nrad cause a broadening of the detector from 17 to 30 channels, *i.e.* there is a need of 38 nrad of vibration per channel broadening. This suggests that even a truck that contributes 29 nrad of vibrations would only contribute about one channel of degradation of detector resolution.

## 5. Motion

The motion of the spectrometer is created by 27 stepper motors controlled by the same IcePAP system (Janvier *et al.*, 2013 ▸) as the rest of the beamline, which ensures a seamless integration into the Veritas control system (Sjöblom *et al.*, 2016 ▸).

### 5.1. Trajectories of optics and detectors

To allow detectors to maintain their position on the Rowland circle and to stay in focus while simultaneously avoiding collision and tension of bellows of the joint mechanics, the motors are run simultaneously through a trajectory setup, where each physical motor is following its own trajectory. The overall setup is orchestrated in the software layer, but each motor driver has an uploaded trajectory file to allow motion to be a function of energy, while speed and position are adjusted (Sjöblom *et al.*, 2023 ▸). Whenever a user changes *e.g.* grating or focus, new tables are uploaded to the drivers to ensure that the system maintains its relative positions.

As the spectrometer motors are linked to each other and drifting would influence any measurement, it is important to avoid long-term drift of the system. Efforts have therefore been invested in optimizing the long-term stability of the positions, as can be seen in Fig. 21 ▸, where six encoders are plotted as a function of time for almost a week. The FWHM of the encoder noise as well as the maximal drift from its average position is included. It can be noted that the detector drift (vertical motion) is 68 nm and even at 4° incidence this would only correspond to a drift on the detector surface by 970 nm, far less than a channel (10.1 µm). Not even the longitudinal drift or the FWHM of the detector table is of the order of a detector channel.

An energy scan is shown in Fig. 22 ▸ with both theoretical predictions (line) and measurements (dots).

The difference between the theoretical position and the actual outcome is of the order of 10 µm FWHM, except for the long translation of the detector tables, which is 230 µm, and the rotation of the detector, which is within 1 microdegree.

### 5.2. Fitting routine for fine adjustments

The correct trajectory of the Rowland geometry is very sensitive to deviations of parameters such as grating radius and line density. However, by finding a few known points along the energy scale, *e.g.* using samples with well known emission, it is possible to fit the key parameters. For this, the control system at the beamline contains a specific Sardana controller that exposes those key parameters for adjusting the detector trajectory, namely detector height offset, detector rotation offset, detector focus offset, grating line density and grating curvature.

Given the recorded detector positions at those known energy points, the routine optimizes the Rowland parameters to generate a trajectory that gets as close to these positions as possible. The output is a set of adjusted parameters that can be entered in the Sardana motor controller.

### 5.3. Motion of spectrometer arm

Before changing the angle of the spectrometer, the entire arm is lifted by a set of retractable air feet, see Fig. 13 ▸(*b*), resulting in low friction for the angular motion.

The angle of the spectrometer arm is changed by a stepper motor connected to a rubber wheel that pushes or pulls the entire spectrometer arm along a curved metal track mounted on the floor of the hall, see Fig. 13 ▸(*b*), lower left corner (Öhrman, 2016 ▸). The motor, gearbox, encoder and rubber wheel themselves are located on a small and separate wagon connected to the arm by a steel rod. In this way, the lifting and lowering of the arm does not push/pull against the central spherical bearing, which is aligned with the focal spot of the beamline and defines the entrance arm to the spectrometer.

The rubber wheel creates enough friction with the metal track to overcome the spherical bearing friction and provides the force needed to get the heavy structure moving. The encoder is not connected to the rear axis of the motor but to the rail itself running on a cog belt to prevent issues with rubber wheel slipping on the rail. As it is an absolute encoder, there is no need for a homing procedure after a power failure.

As the spectrometer arm is changing angle, the experimental chamber (Q-chamber) must also rotate, or the vacuum bellows connecting the two systems will break. To avoid complexity in motion, the spectrometer arm has an encoder scale attached to its frame, while the encoder scale reading head is mounted on the Q-chamber. As the position of the Q-chamber is under closed-loop control, whenever the spectrometer arm is in motion, the Q-chamber will follow to maintain its position on the scale regardless of any rubber slipping or if someone pushes the spectrometer arm by hand. If, for whatever reason, the arm and Q-chamber are not aligned, limit and over-travel switches stop the motion of both motors before excessive forces are applied to the bellows.

The angular speed is 7° min^−1^ and a full rotation of 120° takes less than 20 min. Angle variations as small as 0.1° are controlled in stair case motion, see Fig. 23 ▸, and the arm has the ability to return to a position better than a 0.02°, as verified with a laser tracker with a 1 µm resolution. The backlash and the ability to return to a position from the absolute encoder point of view is within a few millidegrees. The angle is calibrated to correspond to the scattering angle in an RIXS experiment.

### 5.4. Motion of gratings

The two gratings are controlled by Pi N470 piezomotors on a three-leg configuration for each grating to create translation and rotation. The position is read by Renishaw 32 bit encoders with 50 nm resolution. Monitoring the encoder readings, the values remain stable over long periods of time, only occasionally shifting 50 nm up or down. The piezos controlling the gratings are only used during commissioning or optimization activities.

## 6. Future work

The instrument presented in this paper is constantly under development and there are plans to expand capabilities and improve performance during its lifetime. In the following, we discuss some of the work that is planned for the near future.

### 6.1. Detector

The current read-out of the delay-line detector is based on constant fraction discriminators to determine the arrival time of the pulses in the delaylines. In the future, a high-bandwidth sampling system will digitize the filtered and amplified pulses, with a trigger system based on MCP events. Curve fitting will be performed to improve the timing of the events, as well as to help with artifact reduction. In the longer term, a CCD, CMOS or LGAD readout with timing capabilities such as the timepix chips might be used.

### 6.2. Polarimeter

For a more comprehensive picture of the scattering event, the polarization of the scattered light also needs to be measured. This can be done (Brookes *et al.*, 2018 ▸; Dvorak *et al.*, 2016 ▸; Chen *et al.*, 2024 ▸) by using a multilayer mirror and Bragg reflection to separate the horizontal polarization from the vertical.

This requires a multilayer mirror optimized for a specific wavelength and reflection angle and a second detector. As the multilayer mirrors are optimized for certain energies, the polarimeter contains a magazine of up to three mirrors. The mirrors can be translated continuously through the beam, splitting it so that one fraction continues to the main detector (unknown polarization) while the other is reflected to the secondary detector (highly polarized). The mirror arrangement and secondary detector can change the reflection angle to give access to a broader band of energies for each multilayer mirror.

The instrument has already been prepared for such a system, as described in Section 3.2.12, Fig. 16 ▸, and the main parts are already installed, except for the multilayer mirrors themselves.

### 6.3. Long gratings

**Figure 6:**
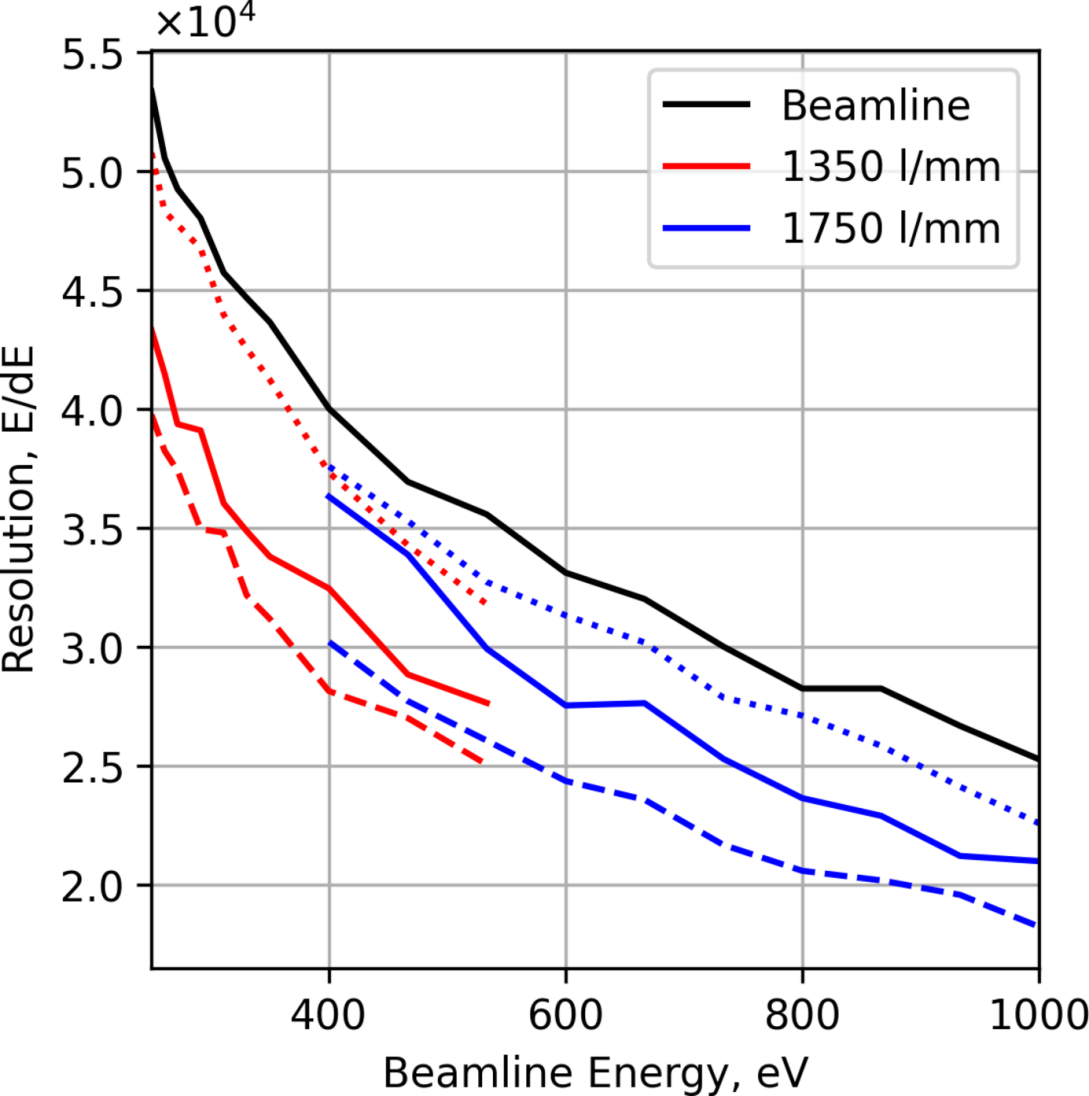
The combined resolution of the spectrometer and the Veritas beamline using the 2400 lines mm^−1^ gratings, 20 m vertical exit slit height and *c*_ff_ = 5. The 1350 lines mm^−1^ (red) and 1750 lines mm^−1^ (blue) gratings are shown over their respective energy ranges for the Pilz (solid), JTEC (dashed) and apertured JTEC (dotted) gratings. Beamline resolution alone shown in black for reference.

**Figure 7:**
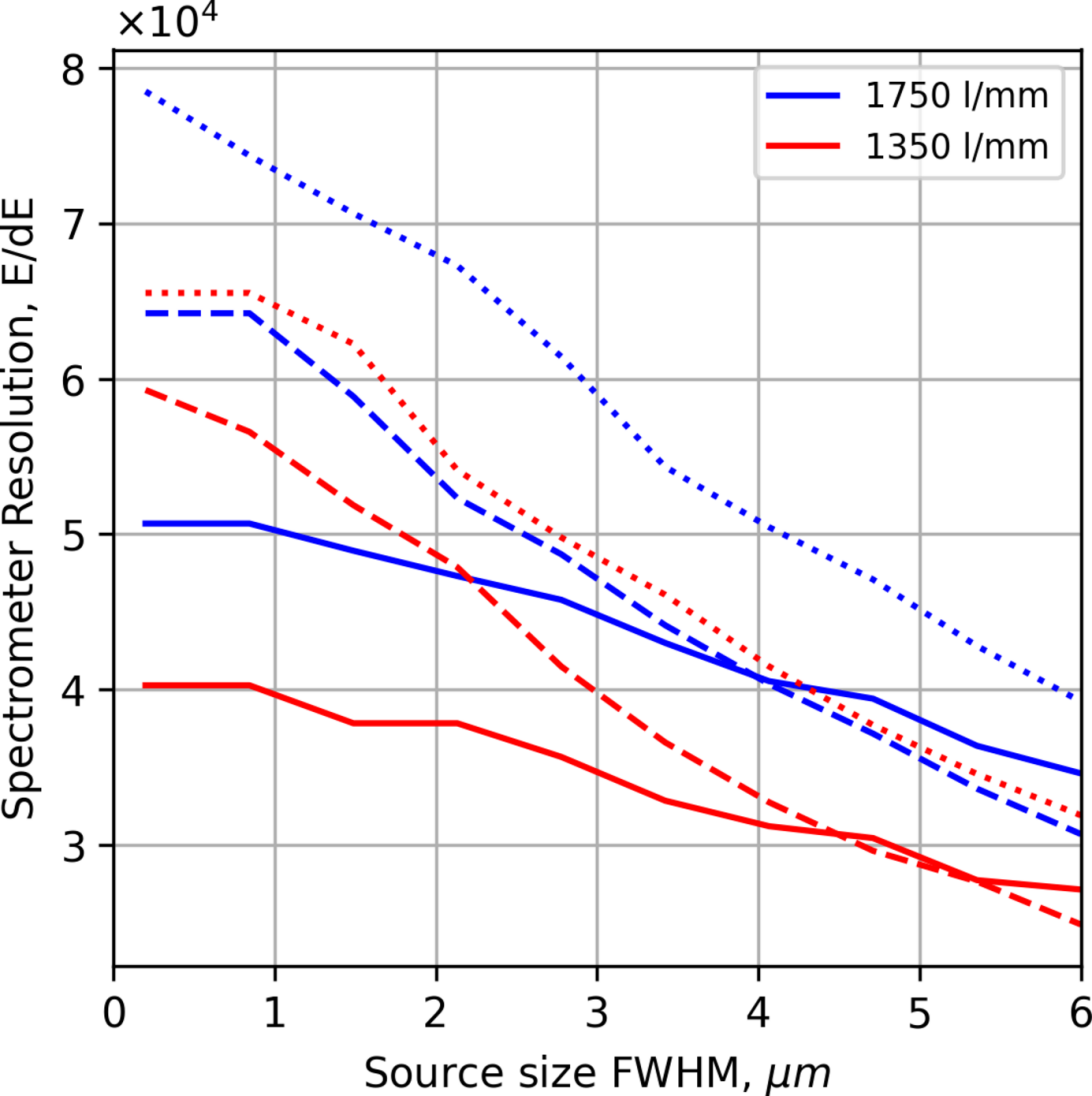
The resolving power of the RIXS instrument for the 1350 lines mm^−1^ (red) and 1750 lines mm^−1^ gratings (blue) at 529.4 eV with varying vertical source size, solid line Pilz, dashed line JTEC, and the dotted line is the apertured JTEC, with a beamline resolving power of 100000.

**Figure 8:**
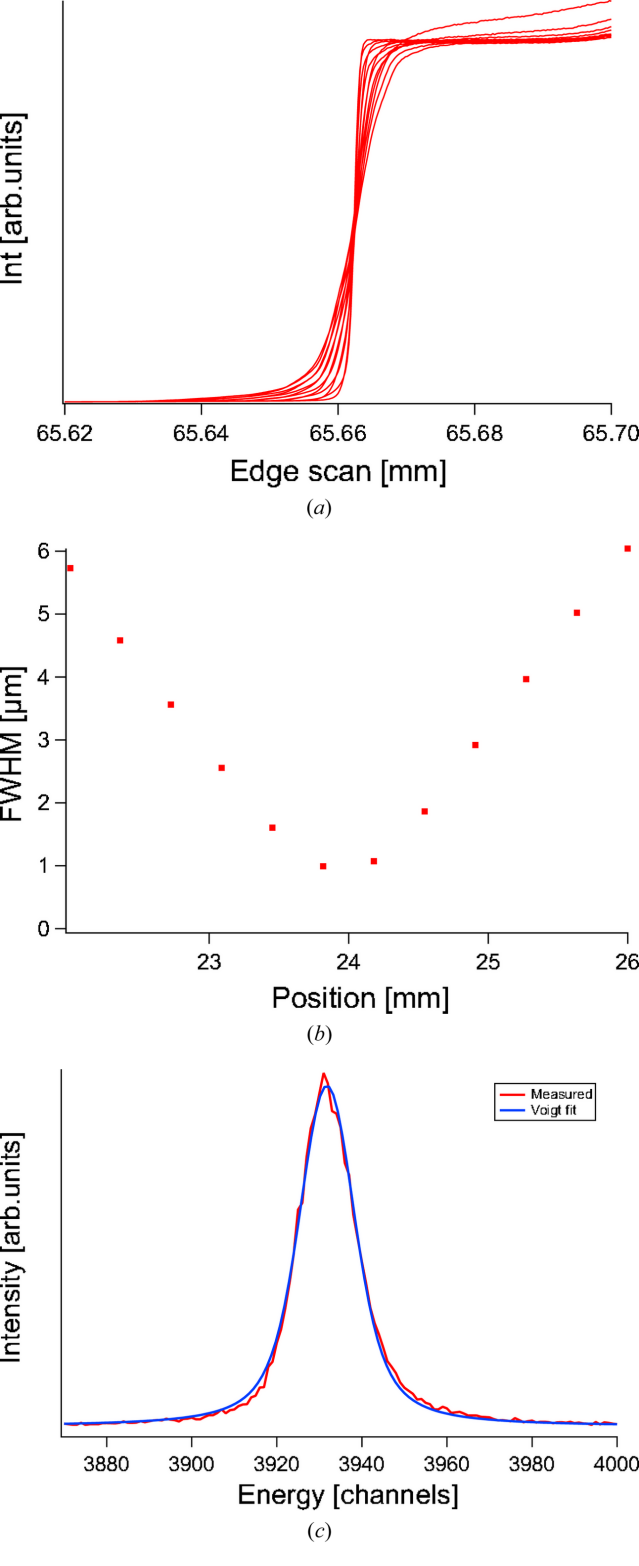
(*a*) Vertical edges scans through the incoming synchrotron beam at different positions. (*b*) FWHM measurements of the vertical focus at different beam positions. Best focus is ∼1 m. (*c*) Elastically scattered light at 468 eV on a 5 m exit slit in the beamline. Voigt fit of the line gives 15.8 channels or ∼158 µm on the detector.

**Figure 9:**
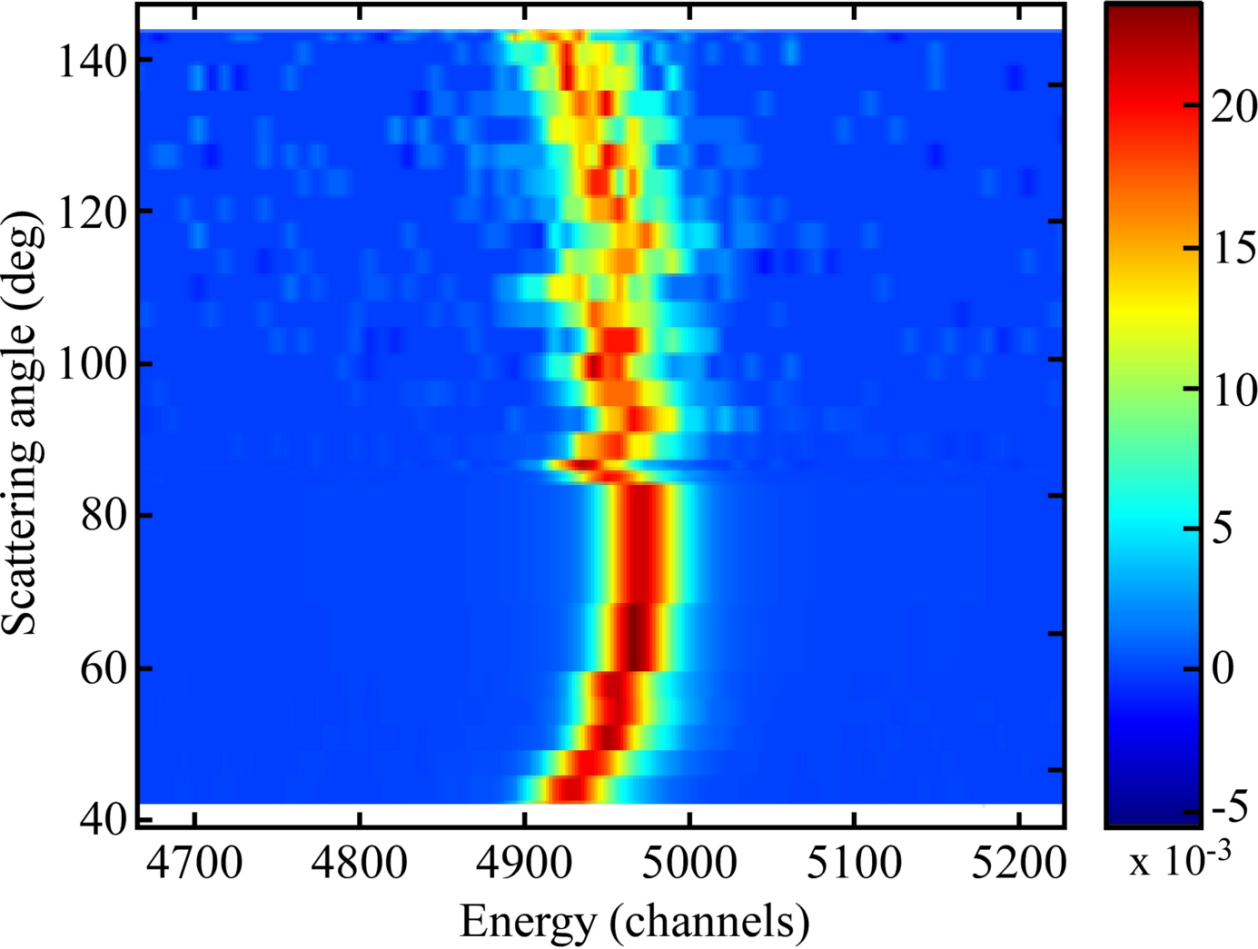
Elastically scattered light at 399.7 eV as the scattering angle (spectrometer rotation) is continuously changed from 42° to 143.2° with a fixed 30° grazing incidence on the sample. At higher scattering angles the intensity of the elastic scattering is quite low which influences the statistics in the measurement.

**Figure 10:**
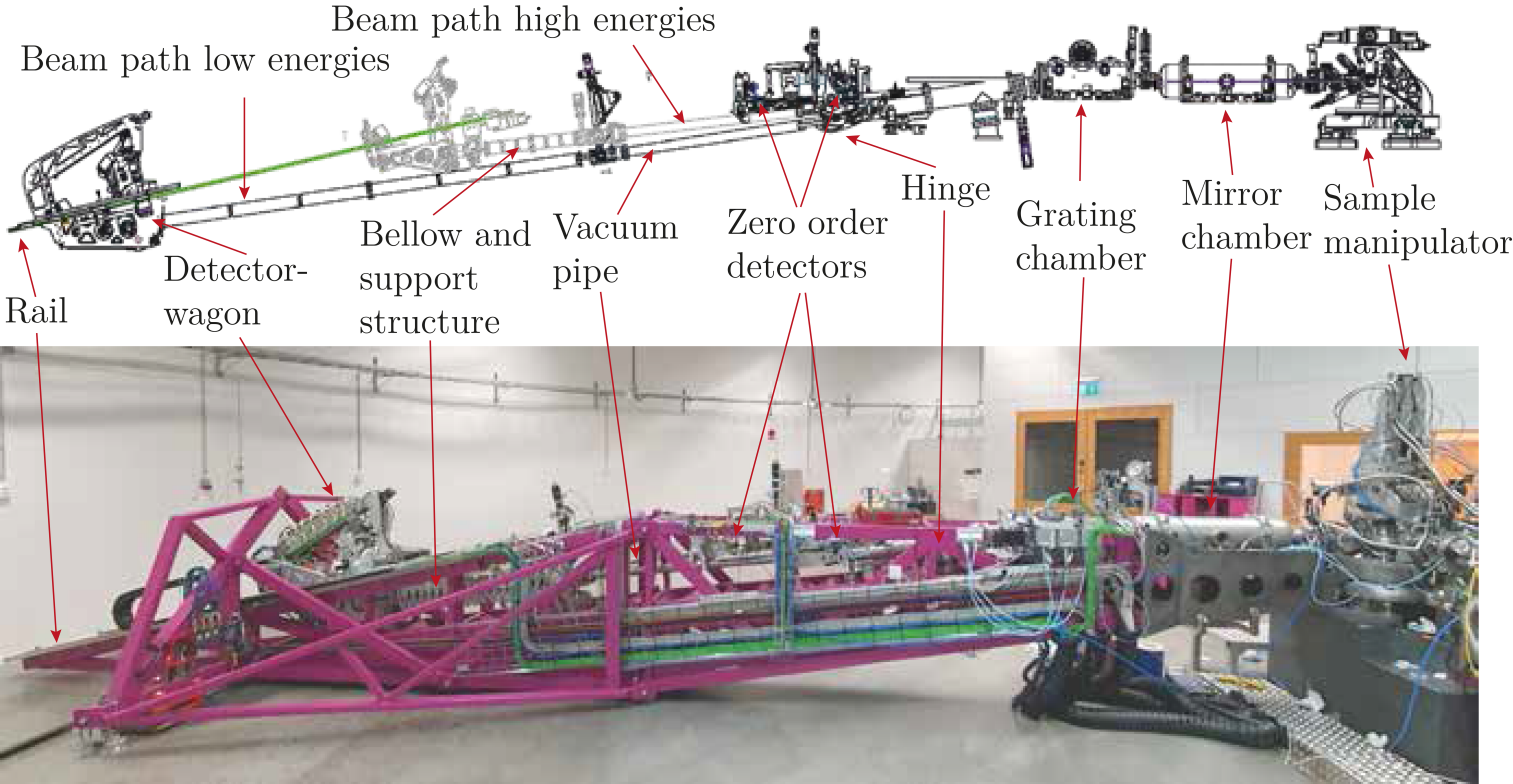
A CAD-model of the spectrometer and its corresponding installation where key features are illustrated.

**Figure 11:**
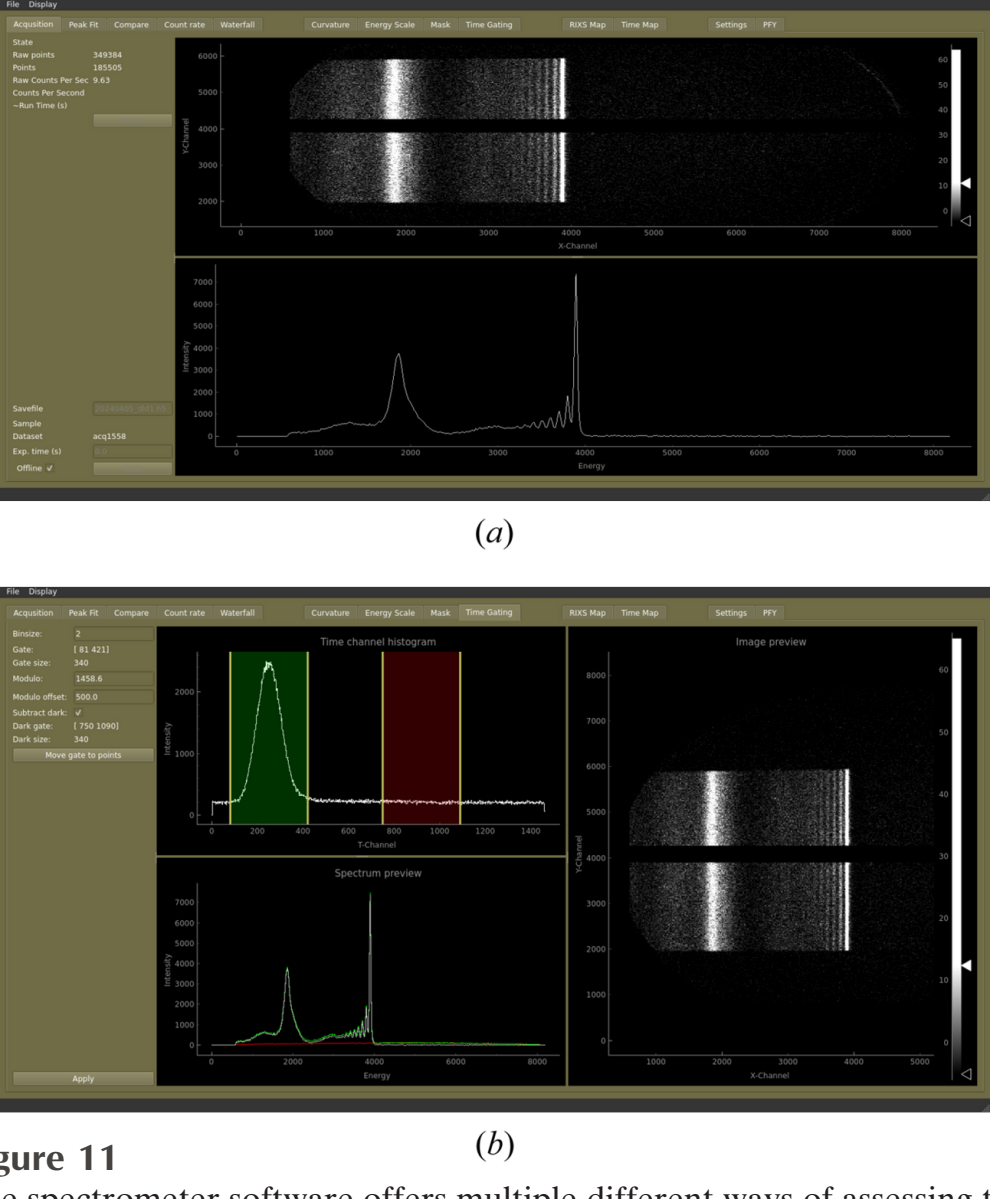
The spectrometer software offers multiple different ways of assessing the measured data.

**Figure 12:**
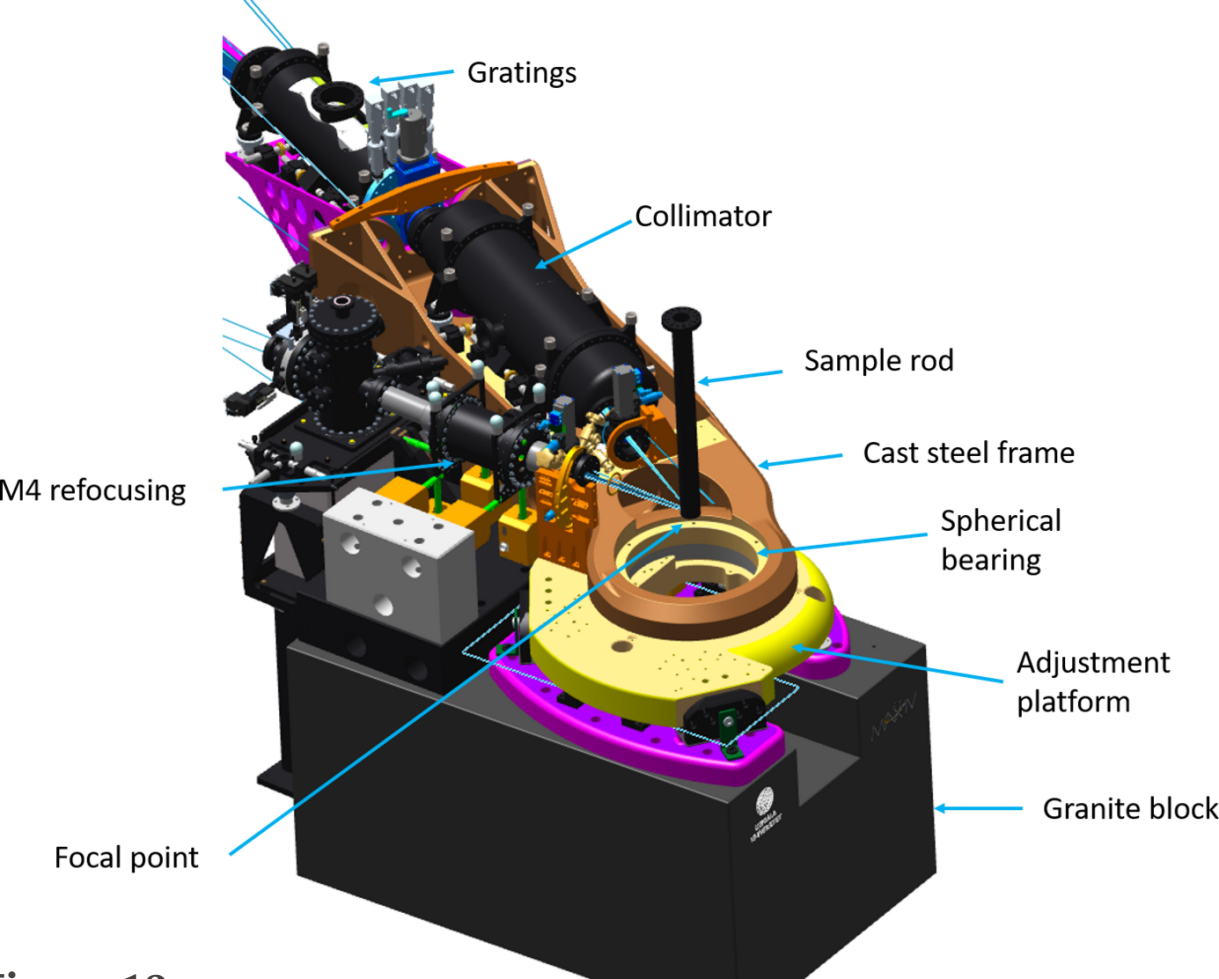
A cast stainless steel frame with optimized strength-to-weight ratio hosts the axial spherical roller bearing making the 120° horizontal rotation possible. The bearing also allows for a small vertical rotation/horizontal tilt created when the arm is lifted prior to rotation in the horizontal plane. The joining structure is a cast stainless steel focal point adjustable platform resting on a 3000 kg granite support. The steel frame houses the collimator and gratings creating a common reference. The focal point adjustment platform also acts as base support for the manipulator frame.

**Figure 13:**
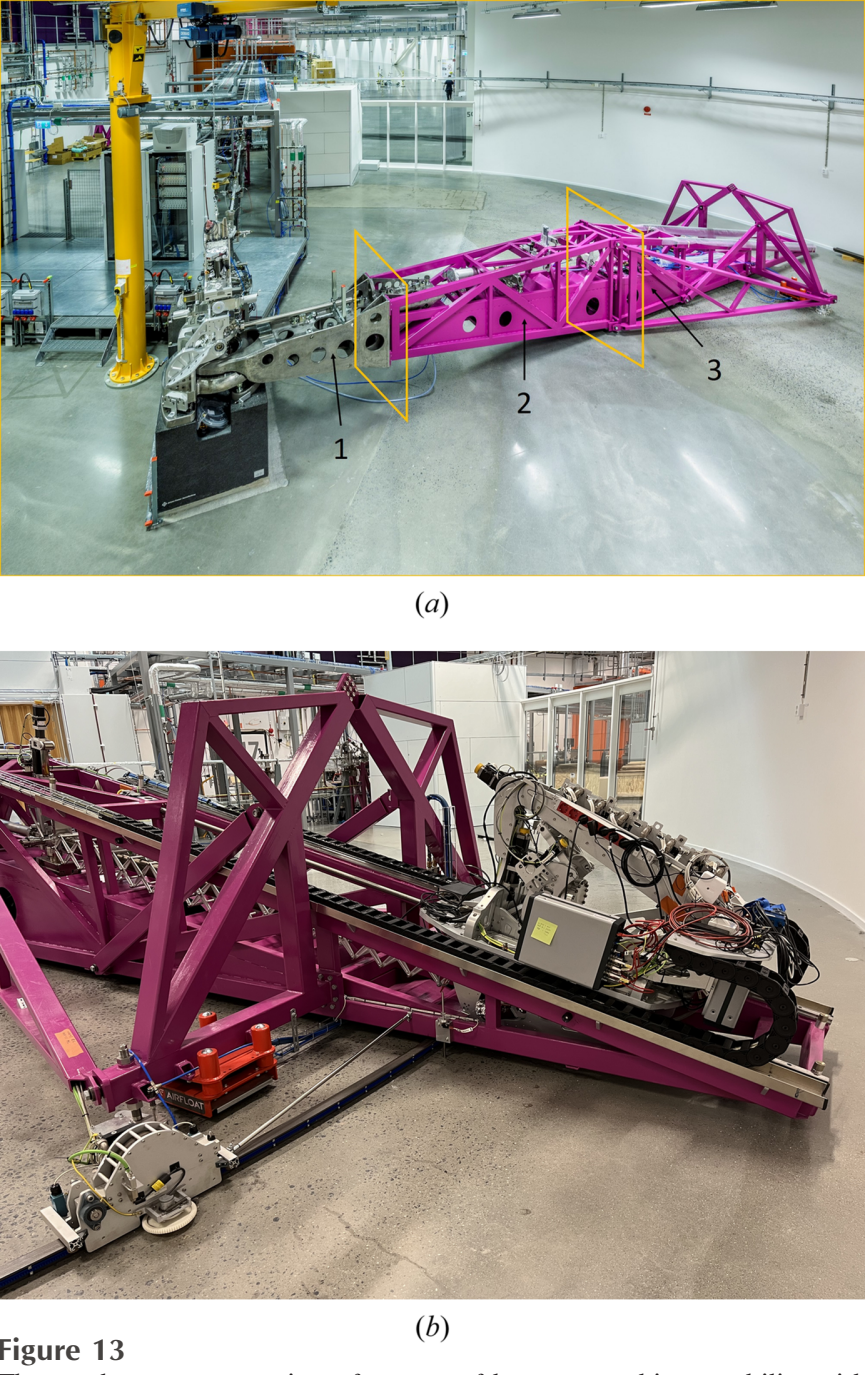
The steel structure consists of a truss of beams to achieve stability with minimal weight. The view from the side (*a*) illustrates the aim to achieve stability by reducing the height above the stable floor. The three spectrometer main arm sections and their interfaces are numbered 1–3. (*b*) The A-arm structure needed to reduce vibrations in the spectrometer arm. Also visible are the red pneumatic elevation feet. The rail structure for the detector sled can be seen on top of the main frame. At the far left the Q-motion drive unit is seen on its combined guide and drive rail.

**Figure 14:**
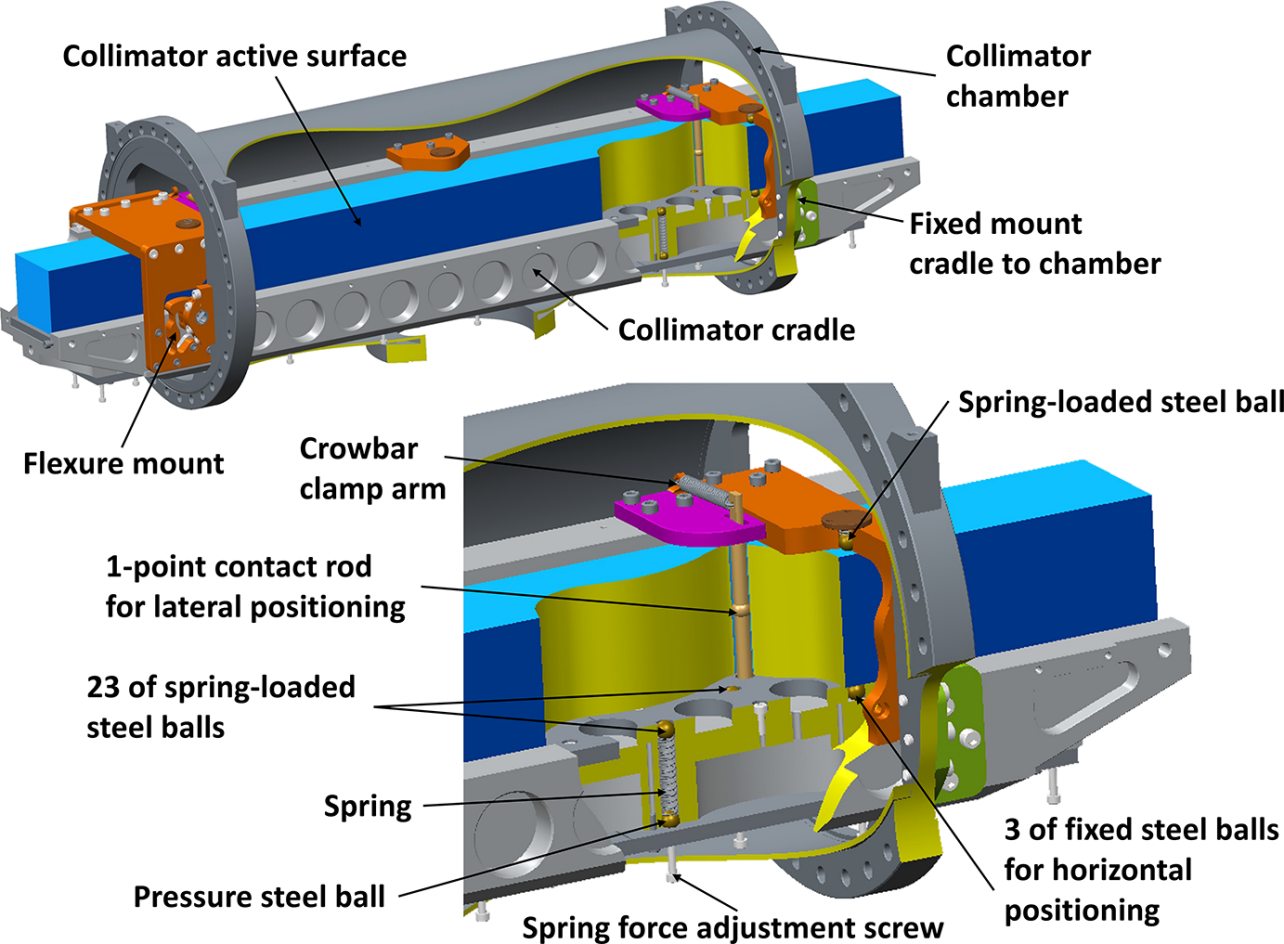
Collimator cradle assembly shown with and without a cutout section illustrating the holding of the collimator.

**Figure 15:**
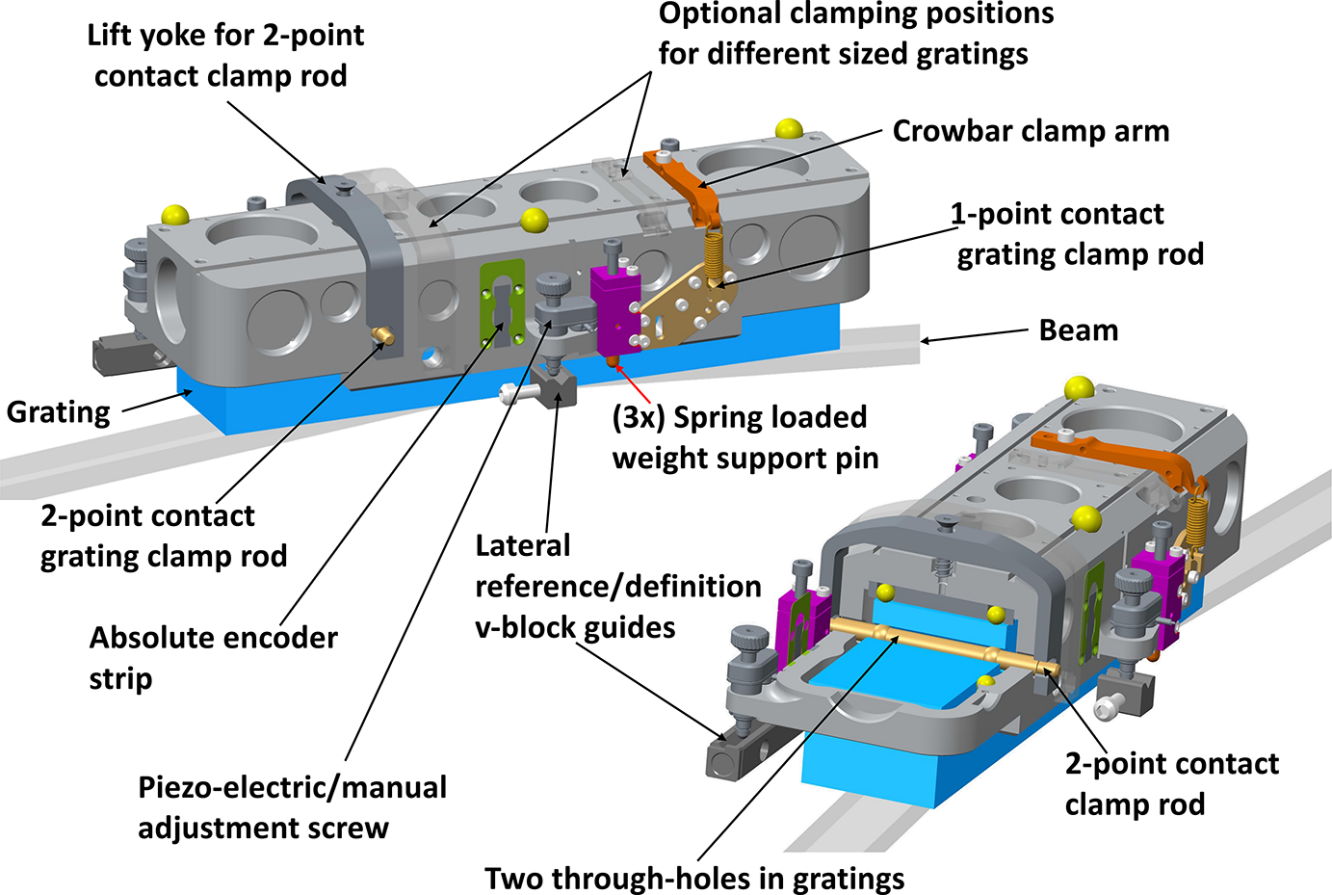
Grating cradle assembly shown with and without a cutout section illustrating the holding of the grating. Also included is the motion concept with three piezo-motors and encoders.

**Figure 16:**
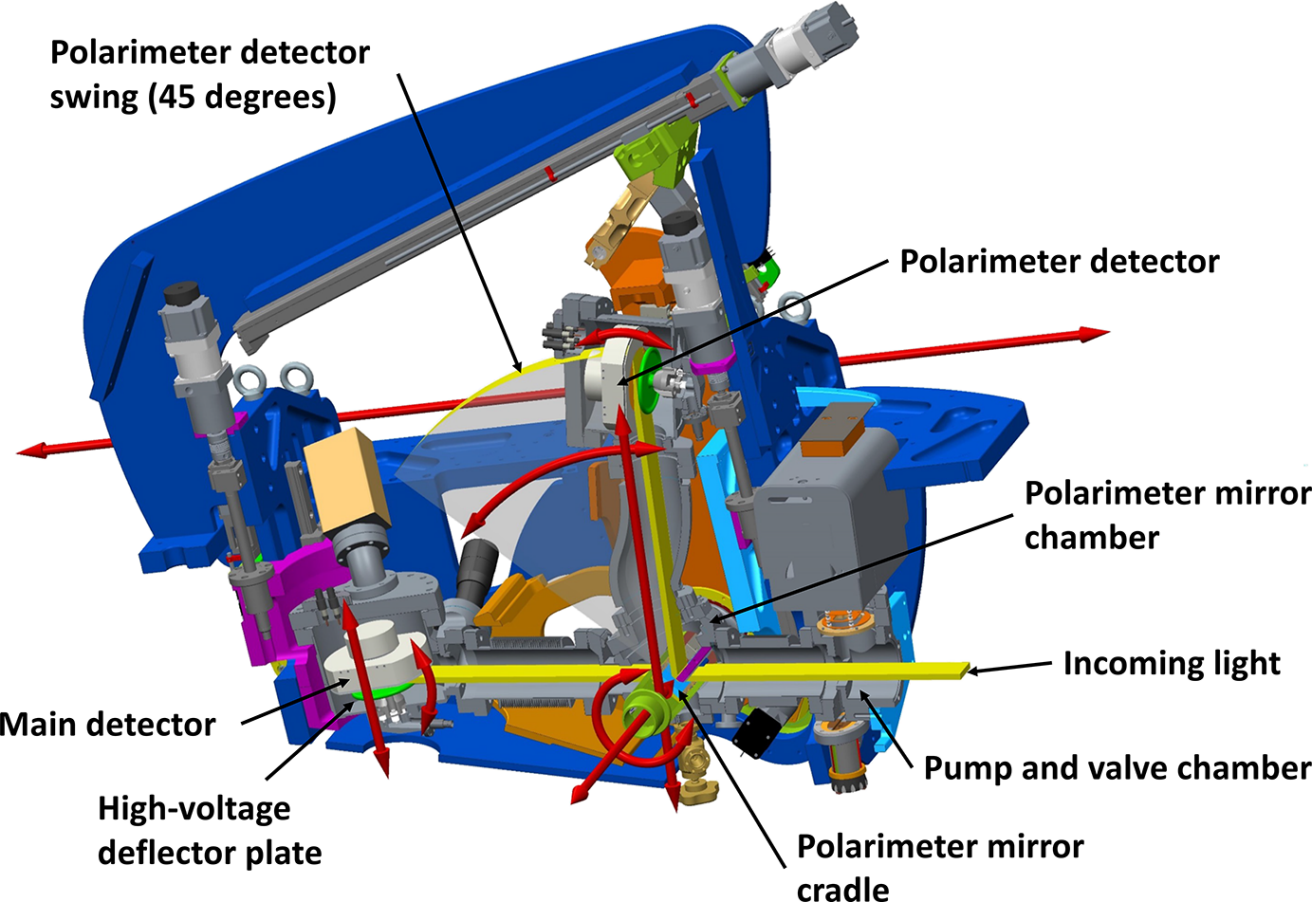
Sled assembly shown with cutout section illustrating the components’ movements and positions.

**Figure 17:**
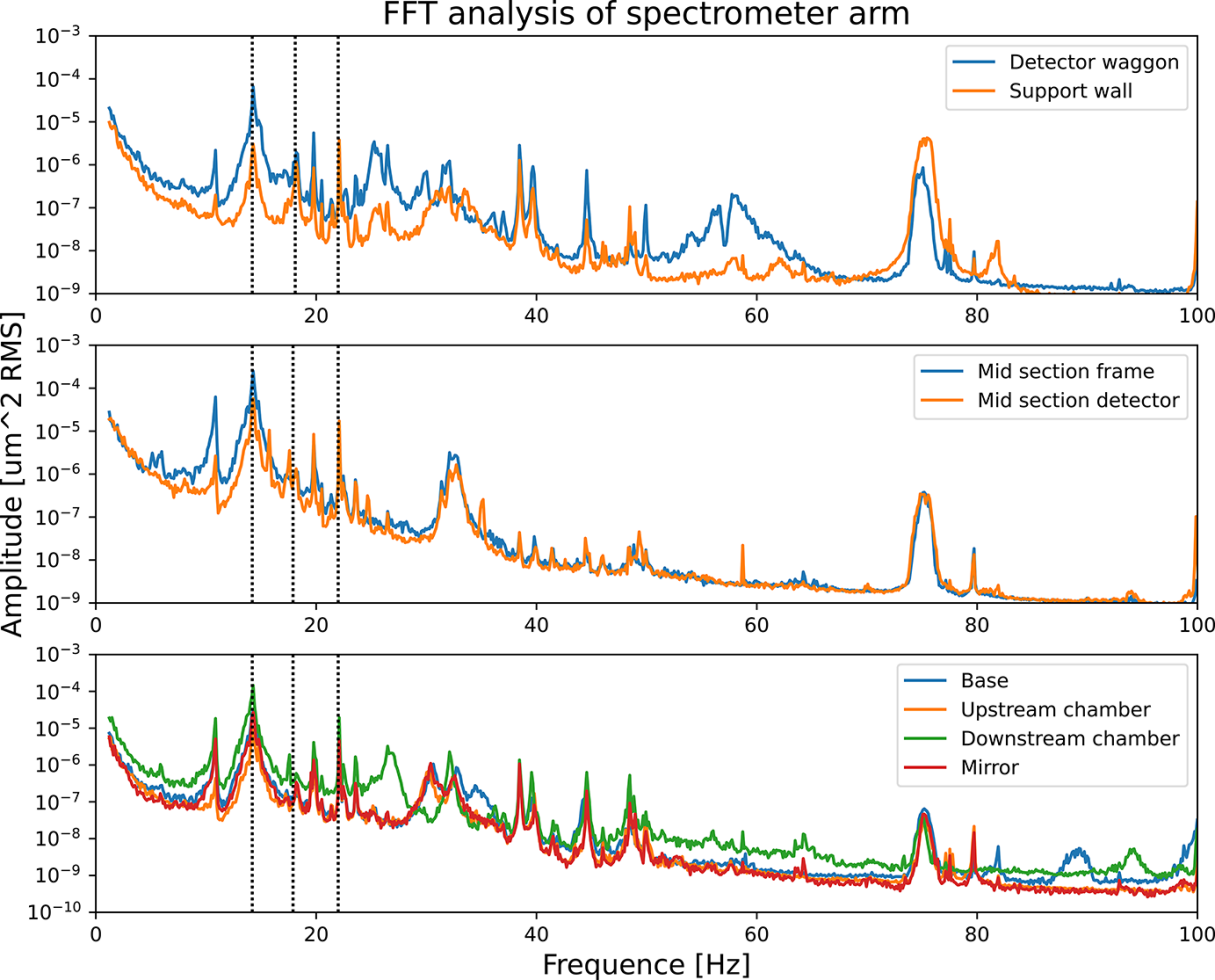
Resonance frequencies as measured with a laser interferometer at three different sections horizontally. Vertical lines indicate the 14 Hz horizontal mode, the 18 Hz vertical mode (also visible horizontally) and the 22 Hz twisting mode.

**Figure 18:**
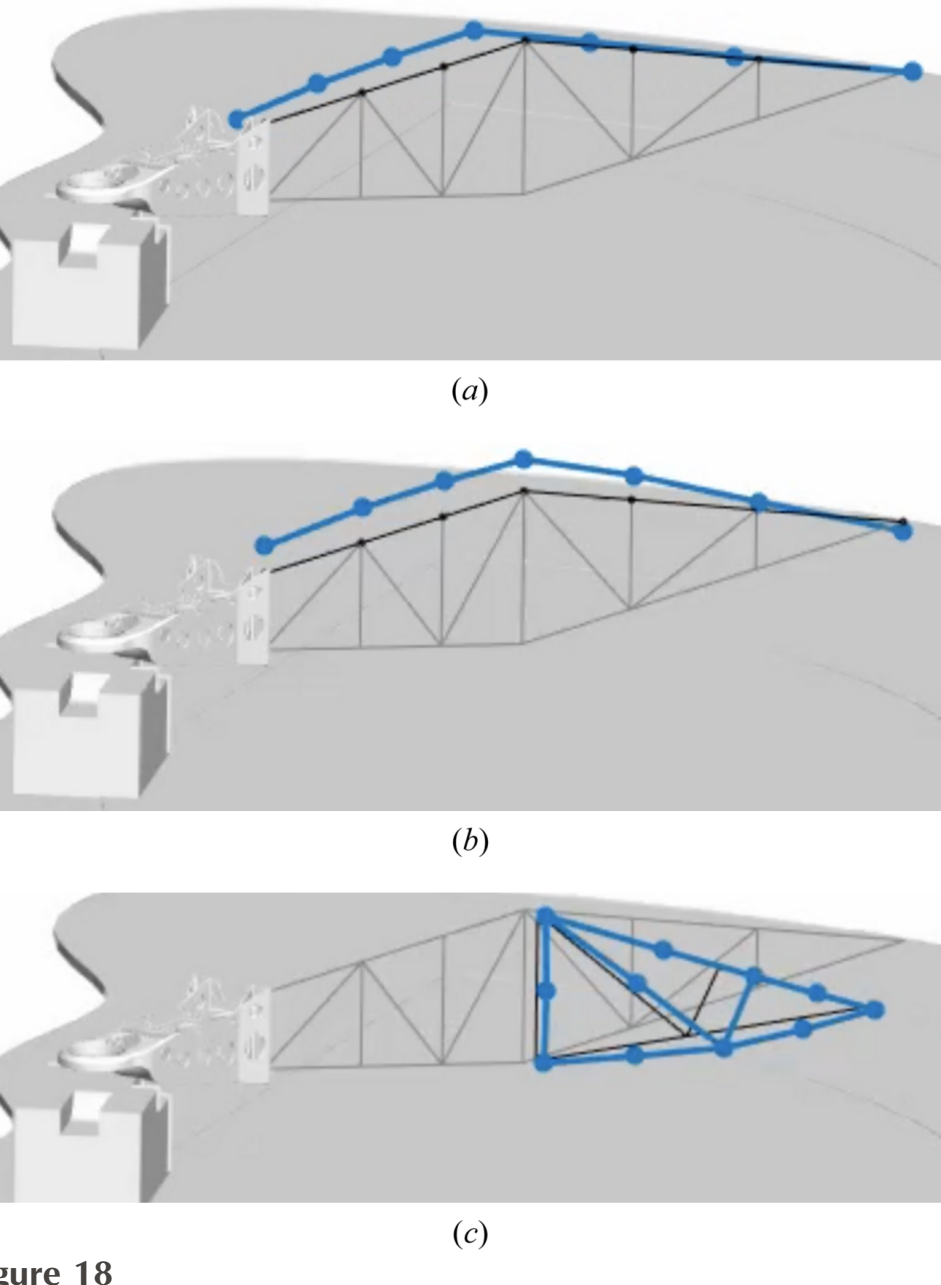
Measured vibration modes in (*a*) 14 Hz horizontal bending mode, (*b*) 18 Hz vertical bending mode and (*c*) 22 Hz twisting mode.

**Figure 19:**
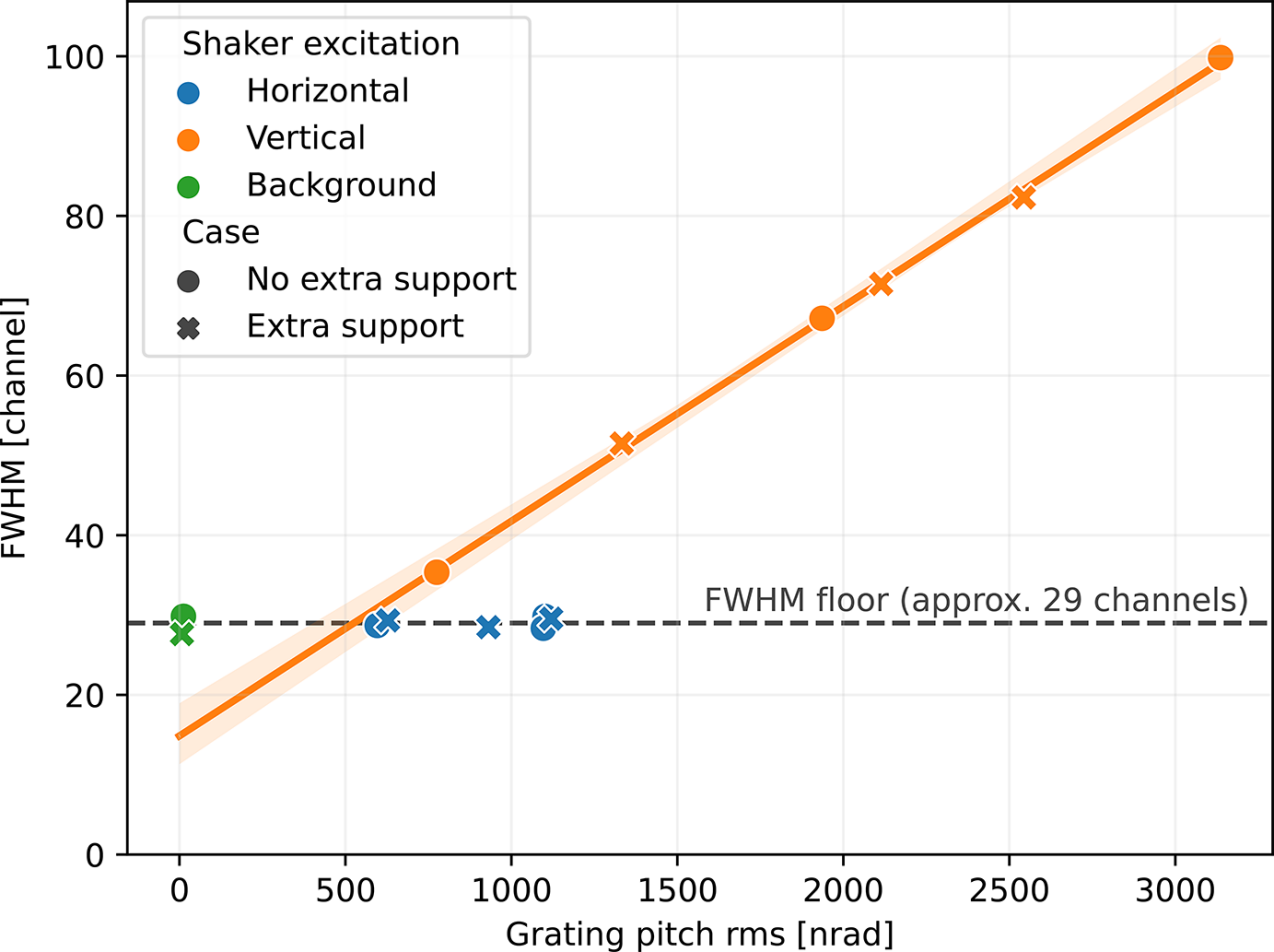
Induced vibration shows a linear relationship between grating pitch and FWHM on the detector. Extrapolating towards zero induced vibrations gives the 17 channel wide image by the detector. Adding extra support under the spectrometer arm forces energy into higher modes and, with the same amount of induced energy, causes a further broadening of the signal at the detector.

**Figure 20:**
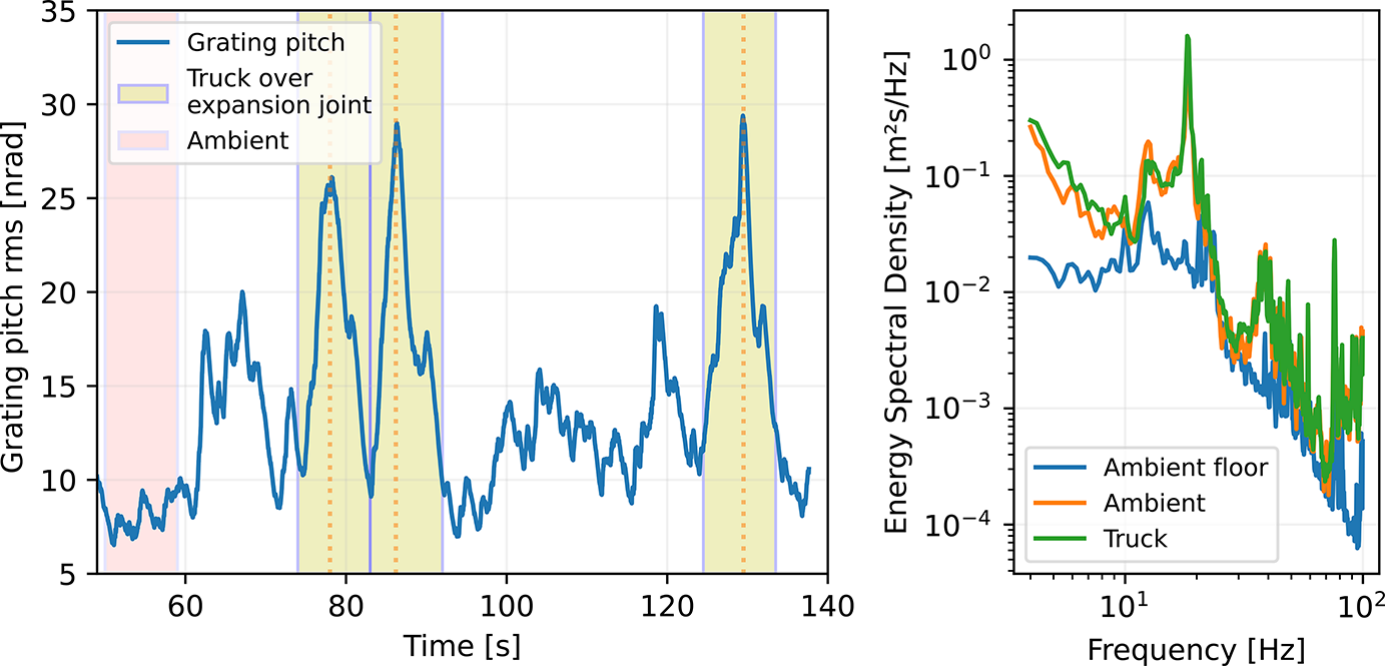
Vibration influence of a nearby expansion joint when a pallet truck passes. Left: vertical vibration peaks due to truck passby. Right: integrated r.m.s. response.

**Figure 21:**
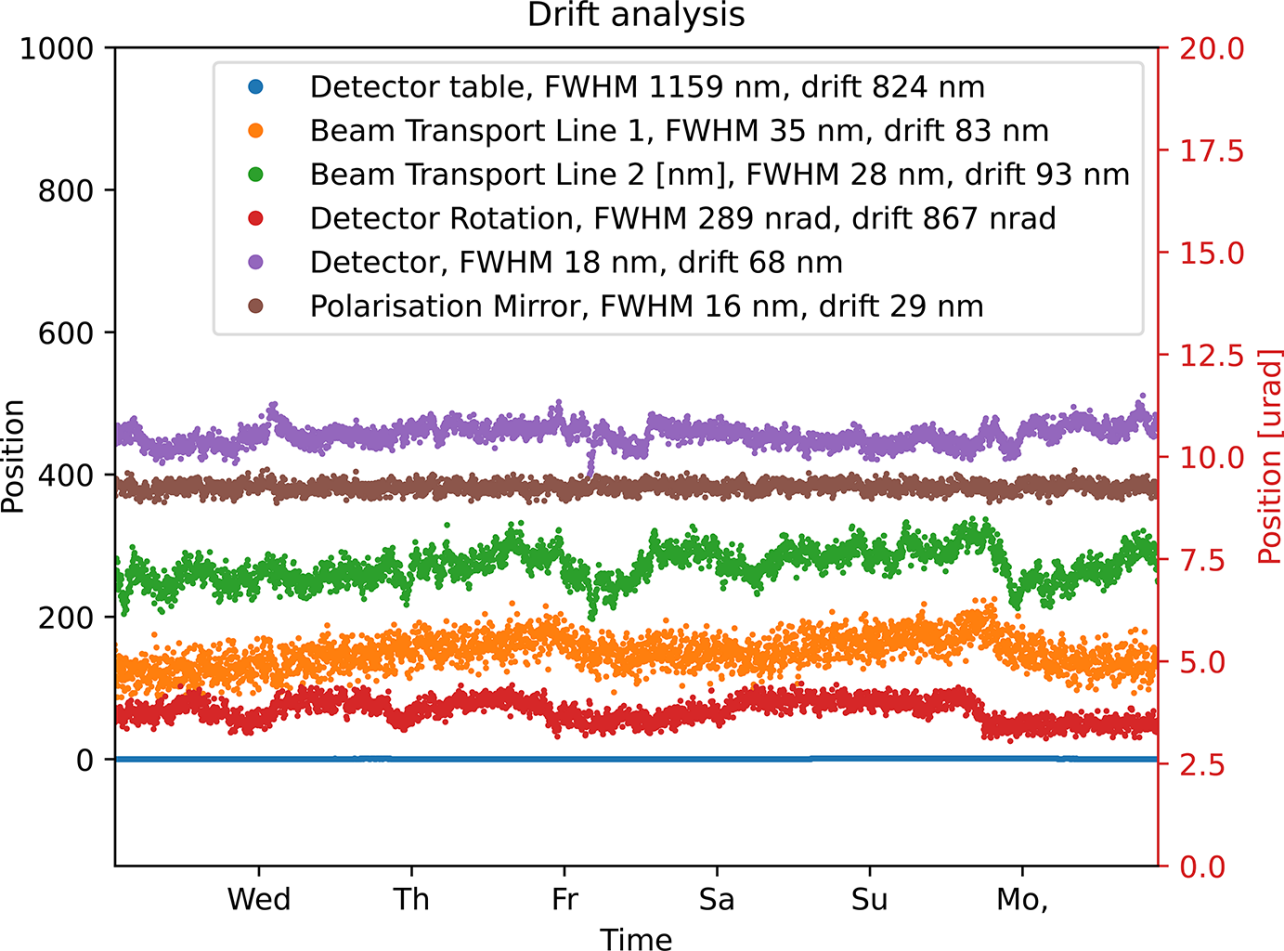
Position is maintained over long time for the motors involved in the parametric trajectories.

**Figure 22:**
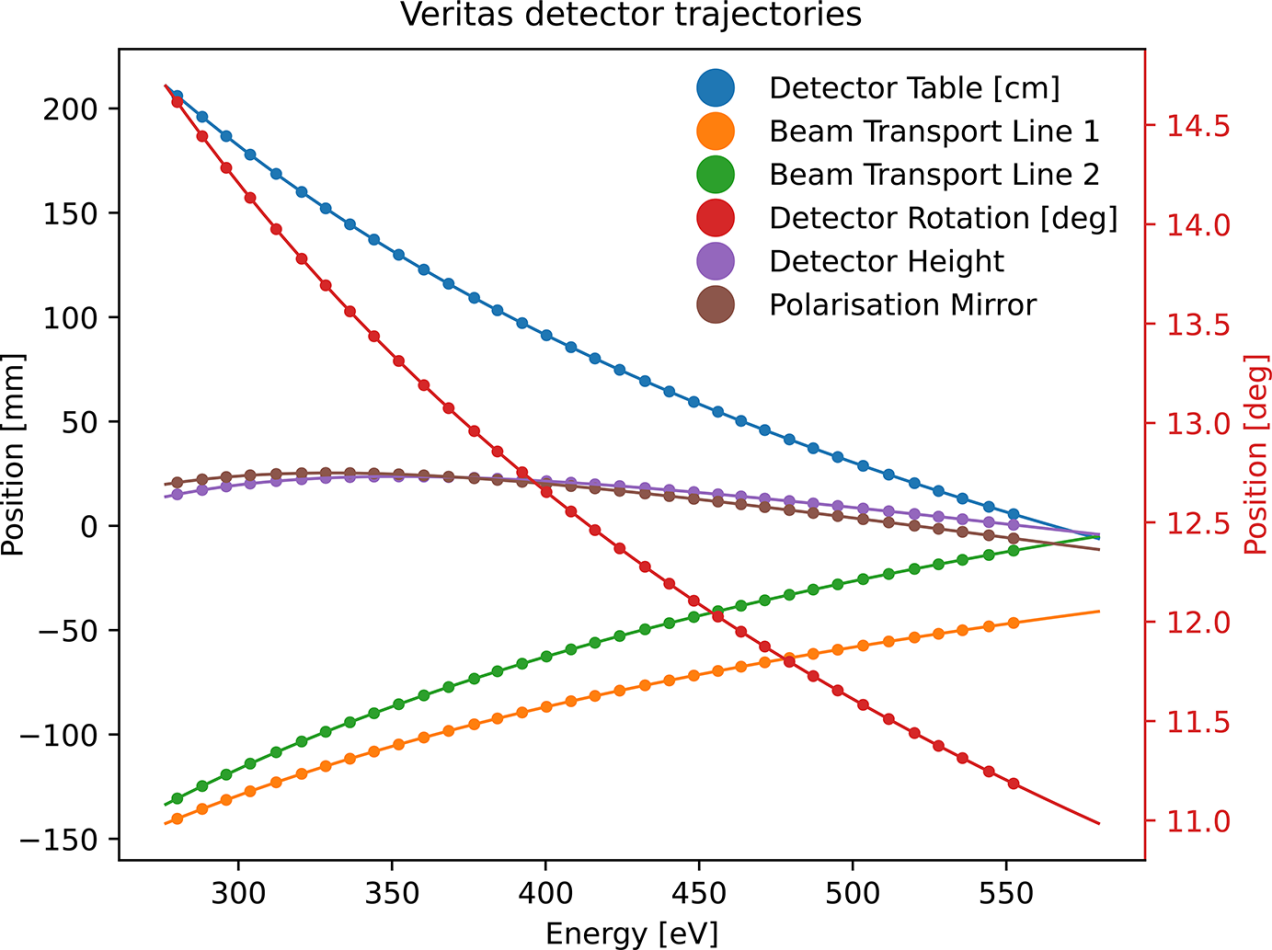
Theoretical calculated position (line) and measured positions with encoder (dots) are compared. The motion closely follows the pre-calculated motion.

**Figure 23:**
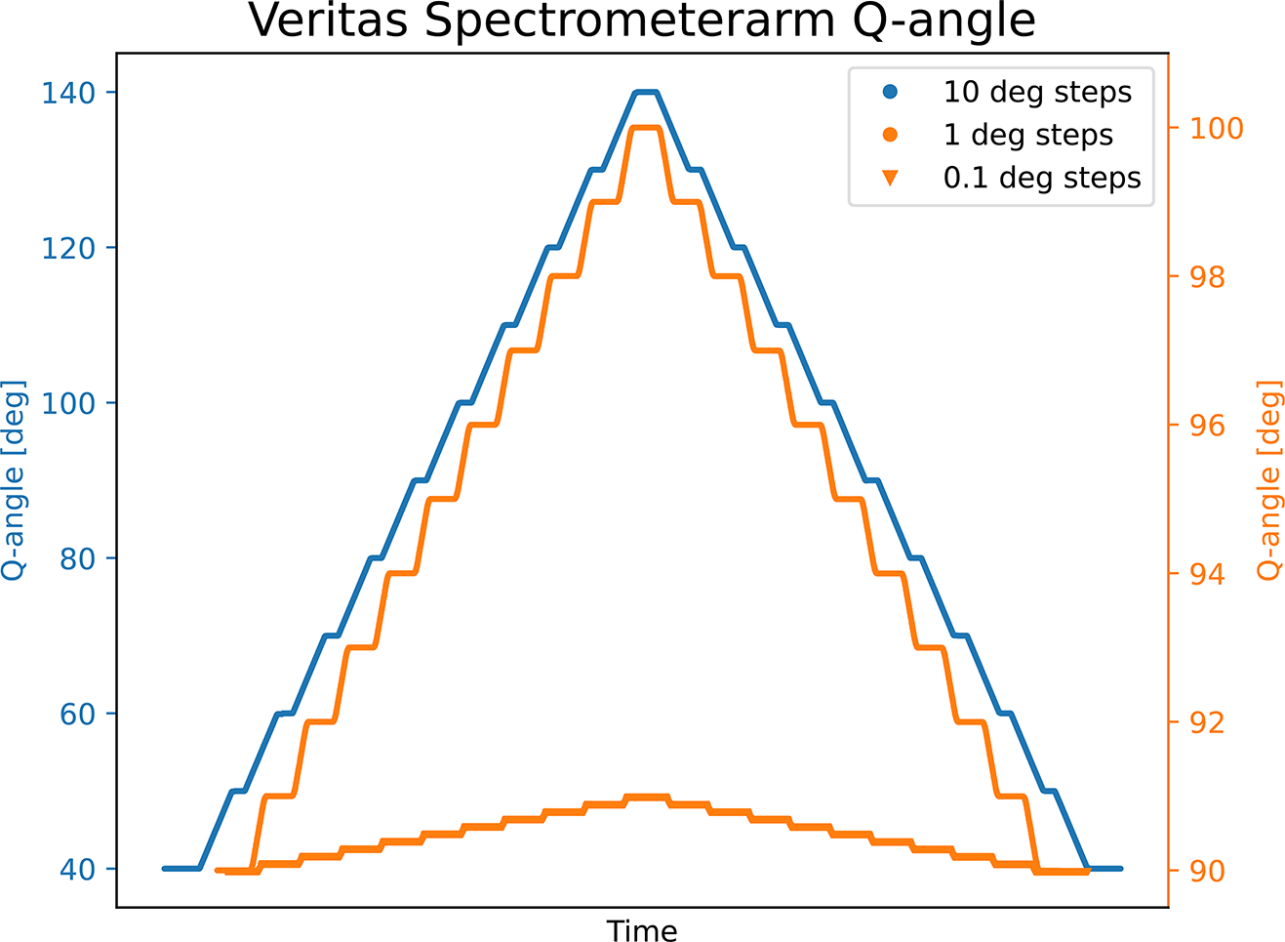
The arm can be moved in a step scan fashion as shown for 10, 1 and 0.1°.

The current performance of the instrument is limited by the surface quality of the installed gratings, named Pilz, and the beamline resolution, as seen in the simulated performance of the spectrometer section[Sec sec2.6]. In Table 1 ▸ there is also a set of high-quality JTEC blanks that have not yet been ruled due to the lack of machines capable of mechanically ruling these larger blanks. Recent developments in ruling technologies, both mechanical (Imprentus, 2025 ▸; HZB, 2025 ▸) and also e-beam lithograph (XRNanotec, 2025 ▸), is opening up the possibility to finish these gratings, which would have a great impact on the instrument performance as long as the beamline performance can also be improved, as seen in Fig. 6 ▸.

## 7. Hardware overview

A short hardware list of key components is presented to provide an overview of the spectrometer, together with key parameters, in Table 3 ▸ showing the current status.

## 8. Conclusion

In this paper, the design and implementation of a collimated Rowland spectrometer for soft X-rays have been presented. The optical design has been evaluated through ray-tracing, showing a potential resolving power of 70–50 K over the energy range of 270 eV to 950 eV. Ray-tracing also shows that the overall performance is closely tied to the beamline performance, and at high transmission (low *c*_ff_) settings of the beamline it is limited by the beamline. Ray-tracing also shows that the slope errors of the optics strongly influence the performance as well as the spherical aberrations, as seen in the comparisons between the two sets of optical gratings/blanks available (Pilz and JTEC).

Although the mechanical design of the instrument has been focused on achieving high eigenfrequencies for critical components, as well as ease of alignment and operation, measurements of vibrational modes present show that there are resonances within the ambient frequency band at MAX IV and that activities close by the instrument can excite modes that are detrimental for the instrument performance. However, because of the specifics of the vibrational modes, critical components such as the optics and detector system are not affected to any large extent, resulting in a negligible impact during normal operation.The complex motions of the instrument as well as of its internal components show great accuracy and long-term stability.

Measurements have so far seen a resolving power limited to around 17 K, which is lower than the design goals. We attribute this to the beamline, which has seen some severe problems with beam stability and energy stability due to problems with mirror heating and cooling. The beamline has generally been operated in a low-resolution mode with high transmission for commissioning and optimization purposes. The high-resolution mode suffers from low transmission and is hard to use for optimization purposes. The sensitivity to the optical component quality is also a concern, as there is currently only one usable set of optics for the instrument, from the same manufacturer, so it is difficult to determine the exact impact of the optical quality.

The spectrometer is now in regular user operation, and further developments at the beamline might result in further improvements.

## Figures and Tables

**Table 1:** Parameters of the optical components—collimator, gratings and polarimeter mirrors (P*x*.*y*)—of the Veritas spectrometer; the optics material is Si(100) coated with 30 nm Au

Optical element	JTEC parabola	JTEC G1	JTEC G2	Pilz G1	Pilz G2	
Size†	1000.8 × 70.15 × 70.2	300.1 × 60.1 × 59.71	300.1 × 59.9 × 60.06	190 × 60 × 45	190 × 60 × 45	
Optical aperture†	955.0 × 35	284.0 × 42.0	281.0 × 40.1	150.0 × 40	150.5 × 40	
Radius‡	—	67.0143	67.025	67.2	67.3	
Line density§	—	Not ruled	Not ruled	1349.622	1750.16	
Incidence angle¶	2	Not ruled	Not ruled	1.75	2.05	
Blaze angle¶	—	Not ruled	Not ruled	1.44	1.35	
Sagittal error††	0.120	0.0248	0.0319	0.160	0.034	
Tangential error††	0.058	0.0180	0.0157	0.052	0.043	
Micro-roughness‡‡	0.174	0.159	0.131	0.55	0.38	

Optical element	P1.1	P1.2	P2.1	P2.2	P3.1	P3.2
Size†	40 × 35 × 8	40 × 35 × 8	40 × 35 × 8	40 × 35 × 8	40 × 35 × 8	40 × 35 × 8
Optical aperture†	30 × 30	30 × 30	30 × 30	30 × 30	30 × 30	30 × 30
Radius‡	−15.84	−14.55	37.8	39.4	>100	69.1
Tangentiral error††	0.060	0.056	0.044	0.047	0.053	0.052
Micro-roughness‡‡	0.3	0.3	0.3	0.3	0.3	0.3

† Dimensions in L × W × T and units in mm.

‡ Units in m.

§ Units in lines mm^−1^.

¶ Grazing incidence at pole in degrees.

†† Slope error unit in arcsec RMS.

‡‡ Units in nm RMS.

**Table 2:** A summary of the specifications of the detectors used in the spectrometer

	DLD 1 and 2	Zero order 1 and 2
Manufacturer	Surface Concept GmbH	Tectra GmbH
Type	Delay-line, MCP	Phosphor screen P43, MCP
Model	DLD 8080	MCP-050-D-S-P43
Purpose	Main experiment	Diagnostic
	Polarimeter	
Active diameter	76 mm diameter	46 mm diameter
Pore size	10 µm	12 µm
Spatial resolution	50 µm	60 µm
Temporal resolution	∼270 ps	50 ms
Coating	Caesium iodide	None

**Table 3:** Hardware overview

Subsystem	Property/device	Unit/dart number
Spectrometer arm	Weight (total) (kg)	3000
	Length (mm)	10000
	Spherical axial roller bearing	SKF 29280
	Lifting feet†	AF01012-4KIT
Mirror chamber	Diameter (mm)	256
	Length (mm)	1100
Grating chamber	Diameter (mm)	206
	Length (mm)	800
	Baffle stepper motors‡	P42H
	Baffle encoders‡	RL32BAT001
	Grating piezomotors§	PI N4470 PT104
	Grating encoders§	RL32BVT050
Bellow	Diameter, inner (mm)	CF63 clearbore 67
	Length min, max (mm)	764, 3305
Detector sled	Weight (kg)	120
	Length (mm)	1170
	Stepper motors[Table-fn tfn10]	PK245/PKP246/PK268
	Encoders[Table-fn tfn10]	RL32BAT001/AGS
	Lead screws[Table-fn tfn11]	BNK1002

† Feet from Airfloat.

‡ Four blade baffle controls illumination of gratings through UHV design, ALBD linear drives with McLennan motors and Renishaw encoders.

§ Each grating has three PI piezomotors located in a triangle to provide rotation freedom and Renishaw encoders to read position.

¶ Several motors from Oriental and encoders from Renishaw. Long motion is with Givi Misure encoder.

†† All are THK brand.
